# Picolinamides with β-Thiophosphorylated Amine Residues as a Useful Scaffold to Generate Biologically Active Pd(II) Pincer Complexes

**DOI:** 10.3390/ijms27083525

**Published:** 2026-04-15

**Authors:** Diana V. Aleksanyan, Aleksandra A. Kalashnikova, Anna Yu. Katranova, Ekaterina Yu. Rybalkina, Nikolay N. Kalitin, Yulia L. Volodina, Yana V. Ryzhmanova, Yulia V. Nelyubina, Oleg I. Artyushin, Zinaida S. Klemenkova, Vladimir A. Kozlov

**Affiliations:** 1 A. N. Nesmeyanov Institute of Organoelement Compounds, Russian Academy of Sciences, ul. Vavilova 28, Str. 1, Moscow 119334, Russia; ma@ineos.ac.ru (A.A.K.); oleg.artyushin@gmail.com (O.I.A.); zklem@ineos.ac.ru (Z.S.K.); fos@ineos.ac.ru (V.A.K.); 2 Faculty of Chemical and Pharmaceutical Technologies and Biomedical Products, D. I. Mendeleev University of Chemical Technology of Russia, Miusskaya pl. 9, Str. 1, Moscow 125047, Russia; 3 N. N. Blokhin National Medical Research Center of Oncology of the Ministry of Health of the Russian Federation, Kashirskoe Shosse 23, Moscow 115478, Russia; kate_rybalkina@mail.ru (E.Y.R.); f.oskolov@mail.ru (N.N.K.); uvo2003@mail.ru (Y.L.V.); 4 Institute of Biochemistry and Physiology of Microorganisms, Federal Research Center “Pushchino Scientific Center of Biological Research of the Russian Academy of Sciences”, pr. Nauki 5, Pushchino 142292, Russia; ryzhmanova@gmail.com; 5 Advanced Engineering School, ITMO University, ul. Lomonosova 9, St. Petersburg 191002, Russia; unelya@gmail.com

**Keywords:** thiophosphorylated amines, carboxamides, palladium, pincer complexes, cytotoxicity, antibacterial activity

## Abstract

The creation of new potential metal-based therapeutics largely relies on the development of useful ligand scaffolds. In recent years, our research group has introduced thiophosphoryl-functionalized carboxamides as a convenient framework for obtaining biologically active cyclopalladated derivatives. In continuation of these studies, β-(aminoalkyl)phosphine sulfides bearing additional substituents in the ethylene backbone were synthesized for the first time and reacted with picolinic acid to afford a series of new functionalized amide ligands. The latter readily underwent direct cyclopalladation under the action of PdCl_2_(NCPh)_2_ under mild reaction conditions. The resulting *S*,*N*,*N*-complexes were studied for in vitro cytotoxicity against several solid and hematopoietic cancer cell lines, as well apoptosis induction and DNA damage ability, which showed their promising anticancer properties. In addition, moderate antibacterial activity was observed for a representative palladocycle of the β-thiophosphorylated derivatives.

## 1. Introduction

**Figure 1:**
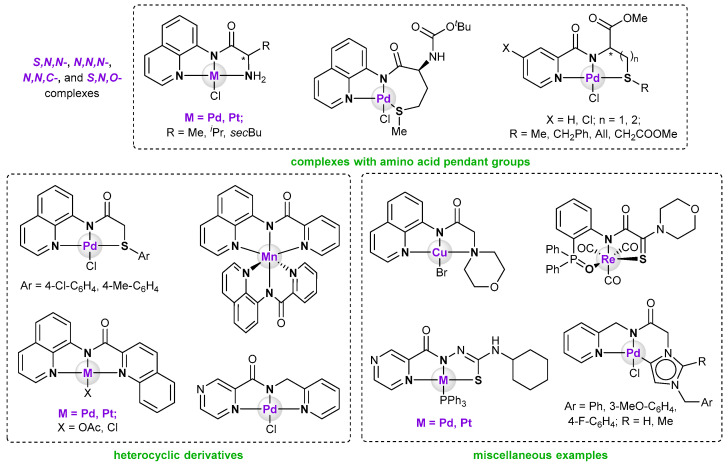
Cyclometalated derivatives of functionalized amides with cytotoxic or antibacterial properties.

Amide groups serve as the key structural units in different types of biomolecules and natural products, as well as synthetic active pharmaceutical ingredients and pesticides, thus attracting continuous interest of scientists across different fields [1,2,3,4]. At the same time, the coordination chemistry of amide-based ligands occupies a special niche in modern bioinorganic and medicinal chemistry. Metal–amide interactions determine the structural peculiarities and reactivity of some metalloproteins and can underlie the biological effects of coordination compounds of carboxamides. Recently, cyclometalated derivatives of functionalized amides have come into research focus as potent drug candidates (see, for example, Figure 1) [5,6,7,8,9,10,11,12,13,14,15,16]. A rigid deprotonated amide core supplemented by heterocyclic, carbene, amino acid, thioether or other pendant units provided firm coordination of transition metal ions, which is especially important in the case of Pd(II) species, and facilitated the anticancer and antibacterial activity of the resulting complexes. Note that such non-classical pincer-type complexes effectively catalyze a variety of chemical reactions [17,18,19,20,21,22,23,24,25,26] and serve as intermediates in different C–H functionalization processes [27,28,29,30,31,32,33]. However, the presence of the amide backbone makes appealing their further application in drug development, in line with the general trend of growing interest to pincer and related tridentate systems in biology and medicinal chemistry [34,35,36,37].

**Figure 2:**
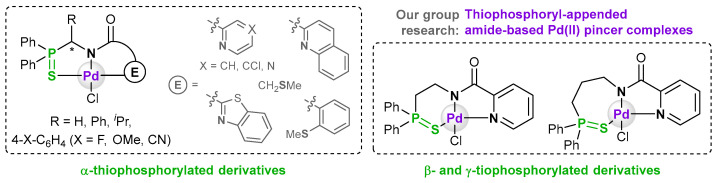
Pd(II) pincer complexes based on the amide ligands with thiophosphoryl pendant arms.

Over the past few years, our research group has devised a series of biologically active Pd(II) complexes of (thio)phosphoryl-appended carboxamides, which were derived from α-(aminoalkyl)phosphine sulfides featuring either substituted or unsubstituted bridging units between the functional groups [38,39,40], and introduced the first examples of their β- and γ-thiophosphorylated analogs with the unsubstituted aliphatic backbone [41] (Figure 2). The structure of the (thio)phosphoryl coordination arm was shown to have a crucial effect both on the stability of a pincer system and biological activity of the cyclopalladated derivatives, where the presence of a sterically hindered amino component promoted higher cytotoxic efficacy. To further examine the bioactivity of this type of Pd(II) complexes, in this work we prepared novel examples of functionalized amide ligands starting from picolinic acid and C1- or C2-substituted β-thiophosphorylated alkylamines, and explored the cytotoxic and antibacterial properties of 5,6-membered palladocycles based on them. The results of DNA damage and cell cycle studies shed some light on the mechanism of their action.

## 2. Results and Discussion

**Scheme 1:**
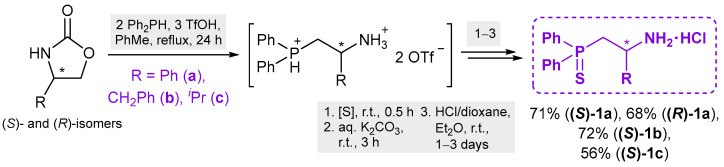
Synthesis of the C2-substituted (alkylamino)phosphine sulfides.

The starting point for obtaining the target functionalized amide ligands was the synthesis of key β-thiophosphorylated amines. In the case of C2-substituted (aminoethyl)phosphine sulfides, a facile route was based on the acid-promoted reactions between Evans’ oxazolidinones and diphenylphosphine followed by the addition of elemental sulfur (Scheme 1). Note that, unlike the protocol initially developed by T. Ikariya et al. [42] for the related amino-functionalized phosphine derivatives, their thiophosphorylated analogs were readily isolated in the form of stable hydrochloride salts, avoiding laborious chromatographic purification under an inert atmosphere. Thus, compounds **1a**–**c** were obtained in reasonably good yields with high purity in gram quantities. The use of commercially available chiral oxazolidinones provided a simple and straightforward access to the enantiomerically pure (aminoalkyl)phosphine sulfides bearing phenyl, benzyl or isopropyl substituents in the backbone. For comparison, both stereoisomers of the phenyl-tethered derivatives were prepared.

**Scheme 2:**
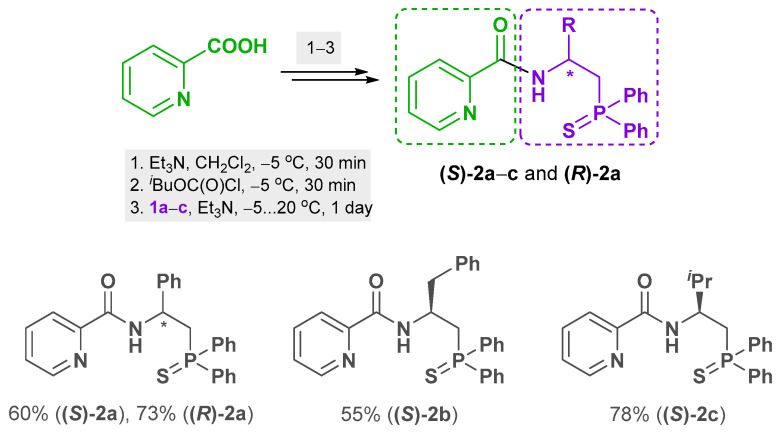
Coupling of β-thiophosphorylated amine derivatives **1a**–**c** with picolinic acid.

Taking into account our previous findings on the effect of the nature of an acid component on the biological activity of amide-based Pd(II) pincer complexes [38,39,40,41], picolinic acid was a primary choice for the coupling with the novel thiophosphoryl-functionalized amine derivatives (Scheme 2). The acid was activated upon treatment with isobutyl chloroformate (IBCF)—an efficient coupling reagent commonly used not only in laboratory practice, but also for large-scale amide bond-forming processes [43], which afforded the desired functionalized amides in moderate to high yields. Compounds **(*S*)-2a**–**c** and **(*R*)-2a** were fully characterized by a set of physicochemical methods, including NMR and IR spectroscopy as well as elemental analysis (for the details, see Section 3 and Supporting Information).

**Scheme 3:**
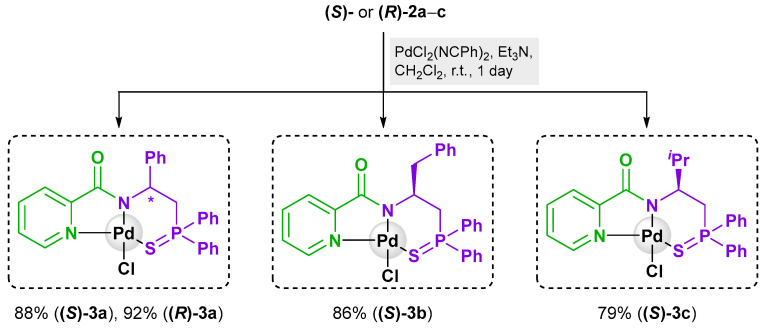
Cyclopalladation of thiophosphoryl-appended amides **2a**–**c**.

The resulting thiophosphoryl-substituted picolinamides were subjected to the reactions with PdCl_2_(NCPh)_2_ under mild conditions in order to provide the desired pincer-type complexes (Scheme 3). The presence of a deprotonable amide core and appropriately arranged pendant groups facilitated smooth cyclopalladation, as was observed earlier for the related systems [38,39,41]. Triethylamine was used as an additive to prevent partial deactivation of the ligands due to the quaternization of a pyridine unit under the action of HCl liberated during metalation. As a result, complexes **3a**–**c** were obtained in high yields.

The realization of κ^3^-*S*,*N*,*N*-coordination in compounds **3a**–**c** was unambiguously confirmed by the characteristic changes in their NMR and IR spectra compared to those of the free amide ligands. Thus, the coordination-induced upfield shift of the phosphorus resonance ranged within 4.7–5.9 ppm, while the displacement of the P=S bond absorption bands to the lower frequency region reached up to 71 cm^−1^. The downfield shift of the CH proton located in the ortho position to the pyridine nitrogen atom (Δ*δ*_H_ = 0.81–0.86 ppm) indirectly indicated the complexation of this heterocyclic unit. Finally, the absence of the NH proton signals in the ^1^H NMR spectra and amide A and amide II bands in the IR spectra provided firm evidence for the occurrence of metalation. The latter was also reflected in strong downfield shifts of the signals of amide (8.0–8.8 ppm) and adjacent pyridine *ipso*-carbon (4.9–5.4 ppm) nuclei in the ^13^C NMR spectra.

**Figure 3:**
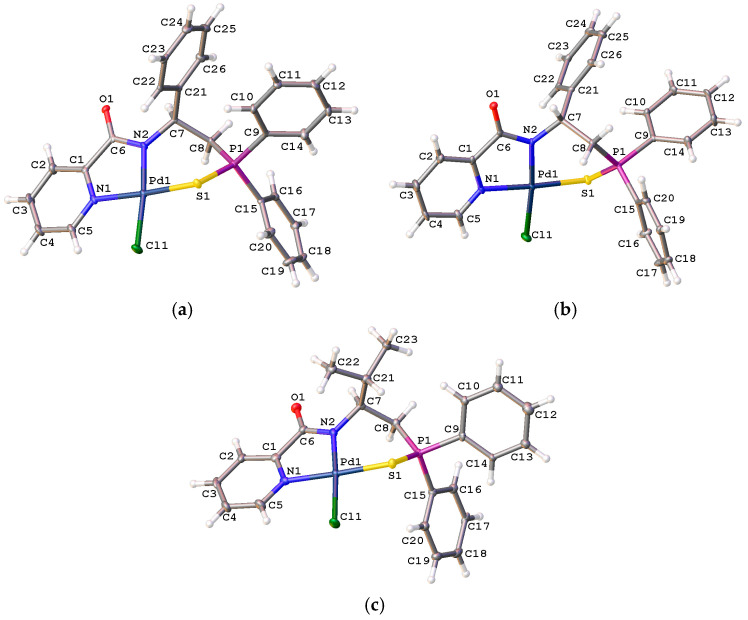
Molecular structures of complexes **(*S*)-3a** (**a**), **(*R*)-3a** (**b**), and **(*S*)-3c** (**c**) in representation of non-hydrogen atoms via thermal ellipsoids at 50% probability level. The minor component of the disorder in **(*S*)-3c** is not shown for clarity.

The X-ray crystallographic analysis of complexes **(*S*)-3a**, **(*R*)-3a**, and **(*S*)-3c** supported the results of spectroscopic studies: in all cases, the palladium atom was found to be bound with the sulfur atom of the thiophosphoryl group, nitrogen atoms of the central deprotonated amide moiety and pendant pyridine unit, as well as the chloride anion (Figure 3). The presence of a mixed donor set inevitably afforded a slightly distorted square-planar geometry around the metal center. However, the main parameters lie in the expected ranges for this type of Pd(II) complexes: Pd–S 2.2930–2.2994, Pd–N(pyridine) 2.036–2.049, Pd–N(amide) 1.999–2.008, and Pd–Cl 2.3061–2.3220 Å. More importantly, the XRD studies confirmed the nonracemic nature of the coordinated ligands and allowed for determining the absolute configurations of the asymmetric carbon centers, which were found to be opposite for complexes **(*S*)-3a** and **(*R*)-3a**.

**Scheme 4:**
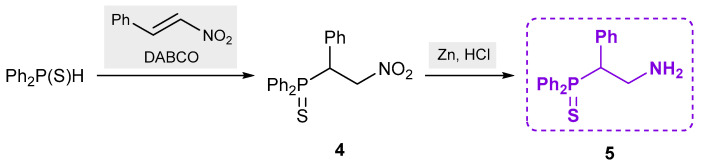
Synthesis of the C1-substituted β-thiophosphorylated amine.

In order to evaluate the effect of a position of the additional substituent in the β-thiophosphorylated amine backbone on the biological profile of the resulting Pd(II) complexes, it seemed interesting to obtain C1-substituted analogs of (alkylamino)phosphine sulfides **1a**–**c**. A convenient approach to a phenyl-tethered derivative was earlier suggested by S.-i. Hirashima et al. [44] and included the addition of diphenylphosphine sulfide to nitrostyrene followed by the reduction of nitro derivative **4** to the corresponding amine (Scheme 4). Although we failed to reproduce the synthesis of compound **5** with the reported efficiency, the target amine was isolated in sufficient quantities for further coupling with picolinic acid. Moreover, despite recent advances in the design of elaborate catalytic systems for the enantioselective synthesis of mono- and disubstituted β-thiophosphorylated nitro precursors [44,45,46], at this point we decided to focus on the production of the target functionalized amide and its cyclopalladated derivative.

**Scheme 5:**
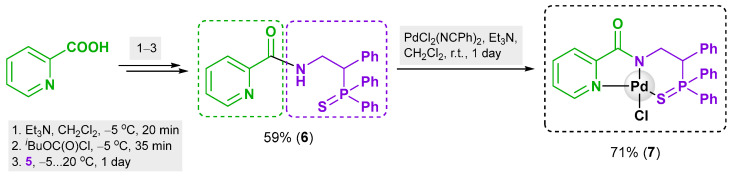
Synthesis of thiophosphoryl-functionalized amide **6** and its cyclopalladation.

The IBCF-mediated reaction of amine **5** with picolinic acid smoothly afforded thiophosphoryl-appended amide **6**, which readily underwent direct cyclopalladation under conditions analogous to the synthesis of complexes **3a**–**c** (Scheme 5). The structures of free ligand **6** and palladocycle **7** were identified using NMR and IR spectroscopy, which revealed the expected spectral changes upon complexation (see Section 3).

To explore the biological activity of the resulting complexes, compounds **3a**–**c** and **7** were screened for cytotoxicity against several human solid and hematopoietic cancer cell lines. The preliminary investigations were performed using the standard MTT colorimetric assay for cellular metabolic activity. The values of half-maximal inhibitory concentrations (IC_50_) achieved in 48 h are summarized in Table 1 and Table 2. The analogous experiments on conditionally normal human embryonic kidney (HEK293) and breast epithelial (HBL100) cells were performed to estimate the selectivity of the tested compounds. Cisplatin from a commercial source was used as a positive control.

In general, the 5,6-membered Pd(II) complexes of functionalized amides derived from substituted β-thiophosphorylated amines exhibited a moderate degree of cytotoxicity on epithelial cell cultures, being inferior to the most active analogs with α-(alkylamino)phosphine sulfide residues (for example, the values of IC_50_ on colon cancer cells HCT116 for the latter ranged within 3.0–7.9 μM) [38,39]. Furthermore, neither the nature of an additional substituent in the amino component, nor the absolute configuration of a stereogenic carbon center had a significant impact on the biological profiles of *S*,*N*,*N*-type palladocycles **3a**–**c** (see entries 1–4 in Table 1). A comparable level of activity was observed against colon (HCT116) and prostate (PC3) solid cancer cells, while breast cancer cells MCF7 appeared to be less sensitive to the compounds explored. Palladocycle **7** bearing a phenyl substituent at the α-carbon atom of the amine residue (entry 5) was slightly outperformed by other complexes obtained in this study in terms of efficiency on HCT116 and MCF7 cells. However, the main drawback of this compound compared to its C2-substituted analogs (complexes **(*S*)-** and **(*R*)-3a**) was the higher toxicity towards non-cancerous HEK293 cells (entries 5 and 1, 2, respectively). The reduced effect on conditionally normal cells (HEK293, HBL100) is a distinctive feature of the complexes under consideration compared to the widely applied anticancer agent—cisplatin. For example, the selectivity indices on HEK293 non-cancerous cells over HCT116 colon cancer cells ranged within 1.4–1.6 for complexes **3a**–**c** and were equal to 0.7 for cisplatin. This difference becomes especially important when considering the results of cytotoxicity studies on hematopoietic cancer cell lines (Table 2). The newly prepared complexes exerted high cytotoxic effects on hemablastosis cells, commensurate with those of the α-thiophosphorylated analogs [38,39], but the selectivity indices of compounds **3a**–**c** were found to be markedly higher, exceeding, as a rule, 3.5 and reaching up to 9.1. Thus, a major outcome of moving from α-thiophosphorylated derivatives to their β-thiophosphorylated counterparts is the enhanced selectivity towards hematopoietic cancer cell lineages. The highest activity of complexes **3a**–**c** and **7** was observed on multiple myeloma cell line AMO1, for which the values of IC_50_ fell into the low-micromolar range (3.6–5.6 μM).

**Figure 4:**
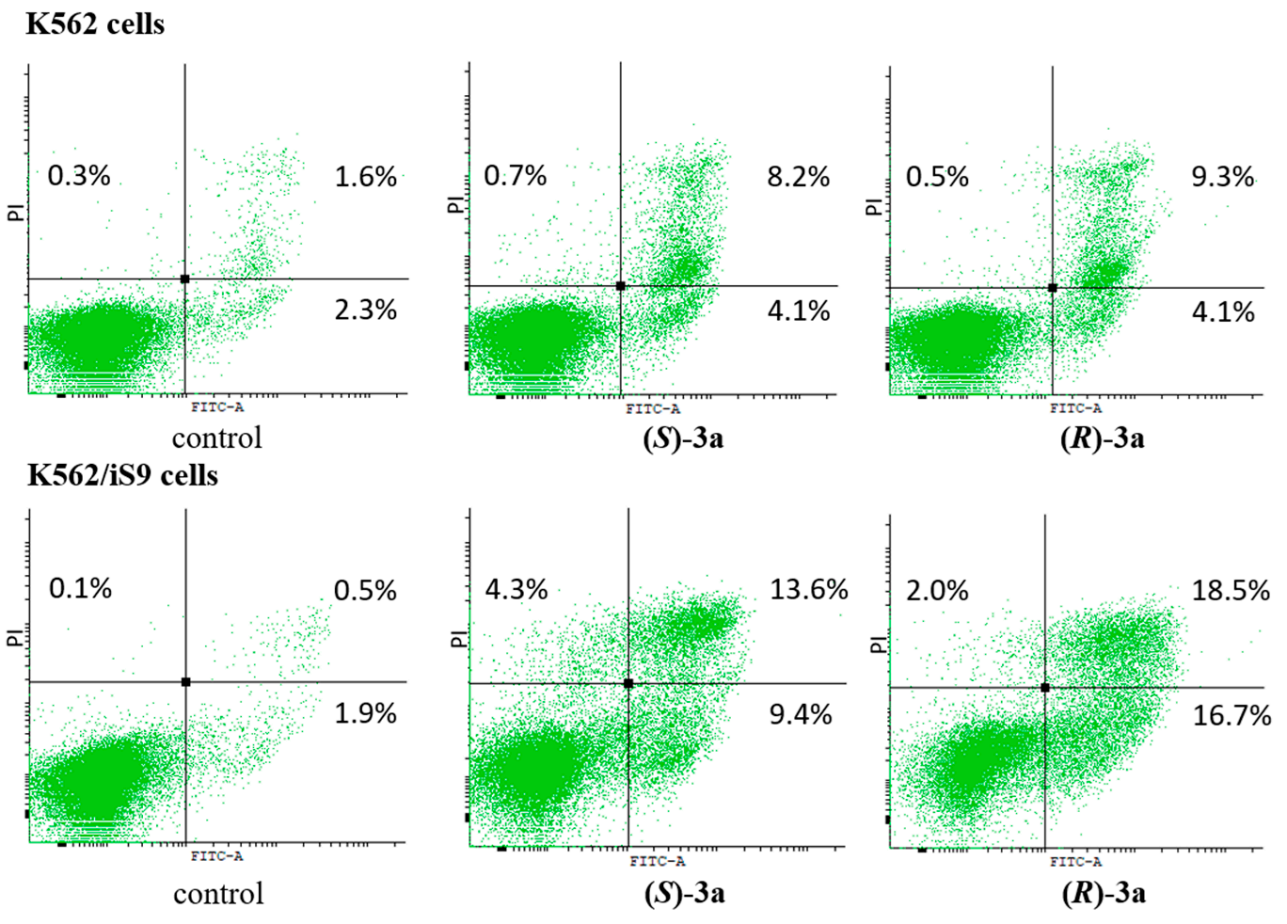
Results of the apoptosis-induction studies: percentages of necrotic (**upper left**), early apoptotic (**lower right**), and late apoptotic (**upper right**) K562 and K562/iS9 cells in the control experiments and after exposure to complexes **(*S*)-3a** and **(*R*)-3a** for 20 h.

In chronic myelogenous leukemia cells K562 and K562/iS9, the resulting *S*,*N*,*N*-palladocycles significantly surpassed in efficiency the clinically used platinum-based drug. Of special note is the comparable or even higher level of activity on the doxorubicin-resistant clones K562/iS9 compared to the parental cells K562, which was further confirmed by the results of apoptosis induction studies of complexes **(*S*)-3a** and **(*R*)-3a** used as representative examples (Figure 4). The total percentage of early (lower right quadrant) and late (upper right quadrant) apoptotic cells for both of the complexes on K562/iS9 subline was higher than in the parental cells. These results are in good agreement with the earlier observed low affinity of the related *S*,*N*,*N*-palladocycles based on α-thiophosphorylated derivatives towards P-glycoprotein [38], which indicates their potential to overcome drug resistance caused by overexpression of this protein.

It should be emphasized that the cytotoxic properties of the resulting complexes are due to the coordination with Pd(II) ions, since free functionalized amides **2a**–**c** and **5** appeared to be nontoxic at concentrations of 30–50 μM and 60 μM in the case of the hematopoietic and solid cancer cell lines explored.

**Figure 5:**
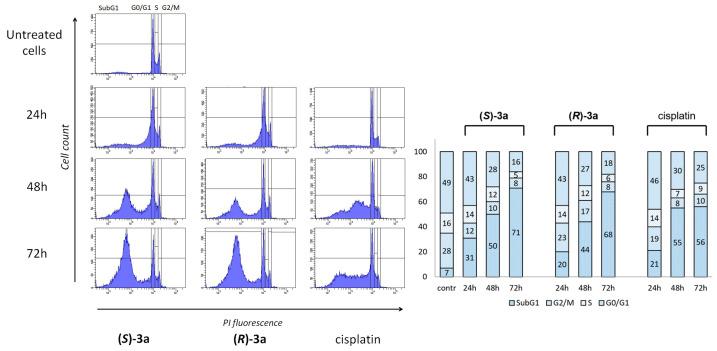
Cell cycle distribution of K562/iS9 cells treated with complexes **(*S*)-3a**, **(*R*)-3a** and cisplatin.

Since cytotoxicity is often associated with the cell cycle disruption, the effects of complexes **(*S*)-3a** and **(*R*)-3a** were assessed by flow cytometry after staining K562/iS9 cell nuclei with propidium iodide to analyze the changes in cell cycle phase distribution. The diagrams depicted in Figure 5 demonstrate a decrease in the G2/M peak relative to the G0/G1 and S peaks after 24 h of incubation with both of the palladocycles. Moreover, after just 24 h of incubation, the number of cells with degraded DNA (dead cells, subG1 peak) increased from 7% in untreated cells to 31% and 20% in the cells treated with complexes **(*S*)-3a** and **(*R*)-3a**, respectively. During further incubation, cell death increased, as is evidenced by a marked growth of the dead cell population (subG1 peak) in 48 and 72 h. This implies that the cells were unable to progress through the S phase of the cell cycle due to the induced disturbances and died.

**Figure 6:**
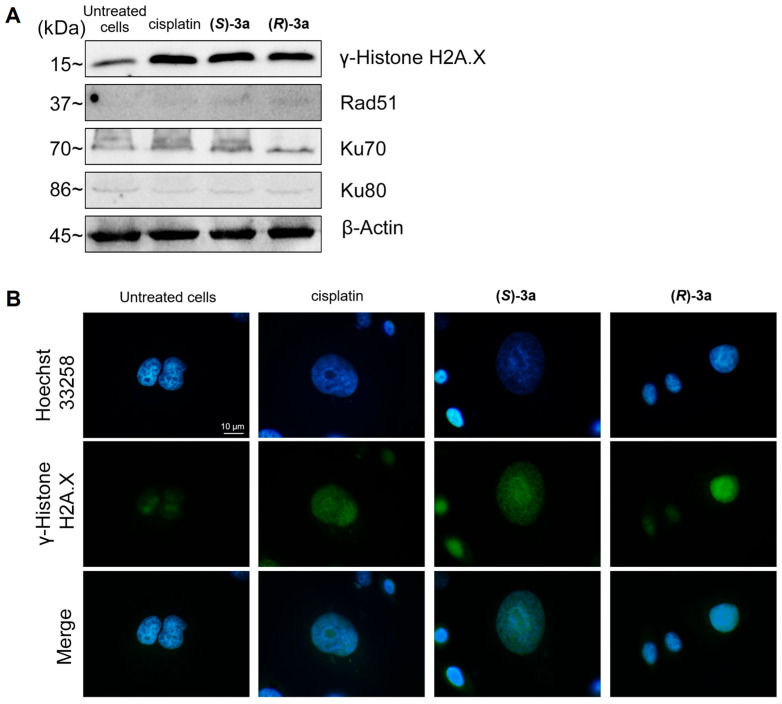
Role of complexes **(*S*)-3a** and **(*R*)-3a** in the induction of DNA damage and propagation of the DNA damage response. (**A**) Effect of the test compounds on the levels of γ-H2AX, Rad51, Ku70 and Ku80 proteins in PC3 cells, as determined by Western blotting. (**B**) Representative images of Rad51 foci induced by compounds **(*S*)-3a** and **(*R*)-3a** in PC3 cells (magnification 100×, scale bar 10 µm).

To further gain insight into the mechanism of action of the compounds under consideration, complexes **(*S*)-3a** and **(*R*)-3a** were studied for their ability to induce DNA damage by Western blotting and immunocytochemical staining of γ-H2AX histone [47]. This phosphorylated form of H2A histone family member X is formed when double-strand breaks appear. As is obvious from Figure 6, the 24 h incubation of PC3 prostate cancer cells with the selected palladocycles led to a sharp increase in the expression of γ-H2AX and the appearance of multiple foci of this histone in cell nuclei, which is comparable to the effect of the well-known DNA-targeting cytotoxic agent cisplatin [48]. The subsequent analysis of the key components of homologous recombination (Rad51 protein) and non-homologous end-joining (Ku70 and Ku80 proteins) systems [49,50], which are required for effective repair of DNA double-strand breaks, did not reveal significant changes in their expression, indicating the lack of activation of the repair system. These preliminary mechanistic findings suggest that the compounds explored may induce strong DNA damage.

Taking into account the growing interest in potential metal-based antibacterial agents, complex **(*R*)-3a** was tested against several Gram-positive and Gram-negative strains using the conventional agar well diffusion method (Table 3). This β-thiophosphorylated cyclopalladated derivative appeared to be moderately effective against all the explored bacterial species at a concentration of 2.50 mM and retained some activity against *Micrococcus luteus* even at a lower concentration of 0.25 mM, while the corresponding free amide (compound **(*R*)-2a**) did not exert inhibitory effects. It is also noteworthy that the yeast *Groenewaldozyma auringiensis* was not sensitive to complex **(*R*)-3a**. The results obtained in this study are somewhat better than those demonstrated by the related α-thiophosphorylated picolinamide derivative [38]. Hence, the biological activity of palladium(II) pincer complexes of thiophosphoryl-functionalized amides has much room for modulation and deserves further investigation.

## 3. Experimental Section

### 3.1. General Remarks

The synthesis of the key amines (except for amine **5**) and amide ligands was carried out under an argon atmosphere. All manipulations for obtaining the target Pd(II) pincer complexes were conducted in the normal atmosphere. Toluene was distilled over sodium under an argon atmosphere prior to the synthesis of the key amine precursors. Dichloromethane was distilled from P_2_O_5_ under an argon atmosphere directly before the synthesis of the target amide ligands and under normal atmosphere for the synthesis of their cyclopalladated derivatives. Triethylamine was distilled over sodium. Thiophosphorylated amine **5** was obtained from the corresponding nitro precursor according to the published procedure [44]. All other chemicals and solvents were used as purchased.

The NMR spectra were recorded on Bruker Avance 300 and Avance 400 spectrometers (Bruker AXS GmbH, Karlsruhe, Germany). The chemical shifts (*δ*) were referenced internally by the residual (^1^H) or deuterated (^13^C) solvent signals relative to tetramethylsilane or externally to H_3_PO_4_ (^31^P). In most cases, the ^13^C{^1^H} NMR spectra were registered using the *J*MODECHO mode; the signals for the C nuclei bearing odd and even numbers of protons had opposite polarities. The NMR peak assignments were based on the data previously reported for the related picolinamide derivatives [38,39]. For the NMR spectra of the representative amine precursors, ligands and their cyclopalladated derivatives, see Figures S1–S15 in the Supporting Information.

The IR spectra were recorded on a Nicolet Magna-IR750 FT spectrometer (Nicolet, Madison, WI, USA) (resolution 2 cm^−1^, 128 scans). The assignment of absorption bands in the IR spectra was made according to Ref. [51].

Column chromatography was carried out using Macherey-Nagel silica gel 60 (MN Kieselgel 60, 70–230 mesh) (Macherey-Nagel, Dueren, Germany).

The melting points were determined with an MPA 120 EZ-Melt automated melting point apparatus (Stanford Research Systems, Sunnyvale, CA, USA).

### 3.2. Syntheses

#### 3.2.1. Synthesis and Characteristics of Amine Precursors **1a**–**c**

**General method for the synthesis.** A mixture of the corresponding (*S*)- or (*R*)-oxazolidin-2-one (1.0 equiv.), diphenylphosphine (2.0 equiv.), and trifluoromethanesulfonic acid (3.3–3.5 equiv.) in toluene (25 mL) was refluxed under an argon atmosphere for 24 h. After cooling to room temperature, elemental sulfur (2.0 equiv.) was added, and the reaction mixture was stirred at room temperature for 0.5 h. Then, a concentrated aqueous solution of K_2_CO_3_, preliminarily degassed by bubbling with argon for 0.5 h, was added dropwise until pH ~ 9, and the mixture was stirred at room temperature for another 3 h. The resulting mixture was poured into a separatory funnel, diluted with Et_2_O (50–70 mL), and washed with distilled water (15–20 mL). The aqueous phase was additionally extracted with Et_2_O (20–30 mL). The combined organic layer was dried over anhydrous Na_2_SO_4_. During separation, the drying agent was additionally washed on a filter with Et_2_O (80–100 mL) to completely remove the trapped product and to dilute the resulting filtrate. Then, a 4 M solution of HCl in dioxane (ca. 1.0 equiv.) was added to precipitate the target amine in the form of a hydrochloride salt. The resulting mixture was left in a refrigerator for 1–3 days, after which the mother liquor was decanted and a new portion of Et_2_O (100 mL) was added, and the resulting mixture was left in a refrigerator for another 1–3 days. In the case of compounds **(*S*)-1a** and **(*R*)-1a**, the target product was collected by filtration, rinsed with Et_2_O (100 mL), and dried under vacuum. After filtration, the glassware was washed with dichloromethane to give, after evaporation and drying under vacuum, an additional portion of the target products as whitish crystalline solids. The analytically pure samples were obtained by additional drying under vacuum over P_2_O_5_. In the case of compounds **(*S*)-1b** and **(*S*)-1c**, the supernant was decanted, the resulting solid or semi-solid residue was triturated with Et_2_O (100 mL) to give a small portion of whitish powder, which was collected by filtration and dried under vacuum to yield analytically pure samples of the target products as whitish crystalline solids. A major part of the resulting residue was dried under vacuum to give the crude hydrochloride salts with >90% purity. In all cases, the key amines in the form of hydrochloride salts remain unchanged at least for several months upon storage under an inert atmosphere.

**(*S*)- and (*R*)-(2-amino-2-phenylethyl)diphenylphosphine sulfide hydrochlorides 1a**


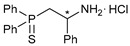


Compound **(*S*)-1a** was obtained by the general method from (*S*)-4-phenyloxazolidin-2-one (1.289 g, 7.899 mmol), Ph_2_PH (2.942 g, 15.801 mmol), trifluoromethanesulfonic acid (3.970 g, 26.453 mmol), elemental sulfur (0.507 g, 15.814 mmol), and 4 M HCl in dioxane (2 mL). Yield: 2.102 g (71%). Mp: >148 °C (dec.). Compound **(*R*)-1a** was obtained by the general method from (*R*)-4-phenyloxazolidin-2-one (1.408 g, 8.629 mmol), Ph_2_PH (3.215 g, 17.267 mmol), trifluoromethanesulfonic acid (4.292 g, 28.598 mmol), elemental sulfur (0.554 g, 17.280 mmol), and 4 M HCl in dioxane (2.3 mL). Yield: 2.197 g (68%). Mp: 191–195 °C (dec.). ^31^P{^1^H} NMR (121.49 MHz, CDCl_3_): *δ* 37.80 ppm. ^1^H NMR (300.13 MHz, CDCl_3_): *δ* 3.30–3.57 (m, 2H, CH_2_P(S)), 4.85 (br. s, 1H, CHN), 7.04–7.13 (m, 3H, H_Ar_), 7.26–7.32 (m, 2H, H_Ar_), 7.37–7.53 (m, 6H, H_Ar_), 7.60 (dd, 2H, *o*-H in P(S)Ph, ^3^*J*_HP_ = 13.4 Hz, ^3^*J*_HH_ = 7.5 Hz), 7.76 (dd, 2H, *o*-H in P(S)Ph, ^3^*J*_HP_ = 13.3 Hz, ^3^*J*_HH_ = 7.6 Hz), 8.98 (br. s, 3H, NH_3_^+^) ppm. ^13^C{^1^H} NMR (100.61 MHz, CDCl_3_): *δ* 35.96 (d, CH_2_P(S), ^1^*J*_CP_ = 56.3 Hz), 52.62 (s, CHN), 128.29 (d, *m*-C in P(S)Ph, ^3^*J*_CP_ = 12.5 Hz), 128.30 and 128.72 (both s, *o*-C and *m*-C in Ph), 128.94 (d, *m*-C in P(S)Ph, ^3^*J*_CP_ = 12.4 Hz), 129.21 (s, *p*-C in Ph), 129.58 (d, *ipso*-C in P(S)Ph, ^1^*J*_CP_ = 81.5 Hz), 130.71 (d, *o*-C in P(S)Ph, ^2^*J*_CP_ = 10.3 Hz), 131.35 (d, *p*-C in P(S)Ph, ^4^*J*_CP_ = 2.5 Hz), 131.50 (d, *o*-C in P(S)Ph, ^2^*J*_CP_ = 10.9 Hz), 131.75 (d, *p*-C in P(S)Ph, ^4^*J*_CP_ = 2.9 Hz), 133.10 (d, *ipso*-C in P(S)Ph, ^1^*J*_CP_ = 85.3 Hz), 134.50 (d, *ipso*-C in Ph, ^3^*J*_CP_ = 4.4 Hz) ppm. IR (*v*/cm^−1^, KBr): 490(m), 512(m), 543(w), 613(m) and 627(m) (both *ν*P=S), 692(s), 713(m), 744(m), 767(w), 862(w), 917(vw), 998(w), 1028(w), 1071(w), 1103(m), 1158(w), 1184(w), 1204(w), 1312(w), 1379(w), 1404(w), 1437(s), 1458(w), 1482(w), 1497(m), 1590(w), 2615(sh, m), 2864(s), 2899(s), 3052(m). Anal. Calcd for C_20_H_21_ClNPS: C, 64.25; H, 5.66; N, 3.75. Found: C, 64.39; H, 5.82; N, 4.01 (**(*S*)-1a**); C, 63.64; H, 5.83; N, 3.66% (**(*R*)-1a**).

**(*S*)-(2-Amino-3-phenylpropyl)diphenylphosphine sulfide hydrochloride (*S*)-1b**


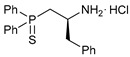


The compound was obtained by the general method from (*S*)-4-benzyloxazolidin-2-one (1.403 g, 7.917 mmol), Ph_2_PH (2.948 g, 15.833 mmol), trifluoromethanesulfonic acid (3.935 g, 26.219 mmol), elemental sulfur (0.508 g, 15.845 mmol), and 4M HCl in dioxane (2.0 mL). Yield: 2.205 g (72%). Mp: 193–197 °C. ^31^P{^1^H} NMR (121.49 MHz, CDCl_3_): *δ* 38.83 ppm. ^1^H NMR (300.13 MHz, CDCl_3_): *δ* 2.84–3.00 (m, 2H, CH_2_), 3.20–3.28 (m, 1H, CH_2_P(S)), 3.59 (br. s, 1H, CHN), 3.67–3.74 (m, 1H, CH_2_P(S)), 7.03–7.07 (m, 2H, H_Ar_), 7.21–7.51 (m, 11H, H_Ar_), 7.60 (dd 2H, *o*-H in P(S)Ph, ^3^*J*_HP_ = 13.5 Hz, ^3^*J*_HH_ = 7.7 Hz), 9.23 (br. s, 3H, NH_3_^+^) ppm. ^13^C{^1^H} NMR (100.61 MHz, CDCl_3_): *δ* 31.06 (d, CH_2_P(S), ^1^*J*_CP_ = 56.5 Hz), 39.59 (d, CH_2_, ^3^*J*_CP_ = 11.3 Hz), 50.61 (d, CHN, ^2^*J*_CP_ = 2.3 Hz), 127.31 (s, *p*-C in Ph), 128.28 (d, *ipso*-C in P(S)Ph, ^1^*J*_CP_ = 80.3 Hz), 128.90 (d, *m*-C in P(S)Ph, ^3^*J*_CP_ = 12.4 Hz), 128.99 (d, *m*-C in P(S)Ph, ^3^*J*_CP_ = 12.2 Hz), 129.05 and 129.64 (both s, *o*-C and *m*-C in Ph), 130.57 (d, *o*-C in P(S)Ph, ^2^*J*_CP_ = 10.4 Hz), 131.44 (d, *o*-C in P(S)Ph, ^2^*J*_CP_ = 10.3 Hz), 132.07 (d, *ipso*-C in P(S)Ph, ^1^*J*_CP_ = 83.6 Hz), 132.12 (d, *p*-C in P(S)Ph, ^4^*J*_CP_ = 2.7 Hz), 132.14 (d, *p*-C in P(S)Ph, ^4^*J*_CP_ = 2.5 Hz), 135.25 (s, *ipso*-C in Ph) ppm. IR (*v*/cm^−1^, KBr): 497(m), 531(m), 609(s) and 621(m) (both *ν*P=S), 691(s), 697(s), 706(s), 746(s), 752(s), 772(w), 798(m), 875(vw), 937(vw), 998(w), 1029(m), 1075(w), 1109(s), 1148(w), 1160(w), 1180(w), 1241(w), 1311(w), 1333(vw), 1362(vw), 1376(w), 1406(w), 1437(s), 1455(m), 1489(br, m), 1561(vw), 1574(w), 1587(w), 1597(w), 2508(m), 2556(m), 2652(m), 2715(s), 2794(s), 2850(m), 2910(m), 2963(m), 3029(m). Anal. Calcd for C_21_H_23_ClNPS: C, 65.02; H, 5.98; N, 3.61. Found: C, 64.96; H, 6.03; N, 3.59%.

**(*S*)-(2-Amino-3-methylbutyl)diphenylphosphine sulfide hydrochloride (*S*)-1c**


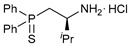


The compound was obtained by the general method from (*S*)-4-isopropyloxazolidin-2-one (0.792 g, 6.132 mmol), Ph_2_PH (2.284 g, 12.267 mmol), trifluoromethanesulfonic acid (3.190 g, 21.255 mmol), elemental sulfur (0.394 g, 12.289 mmol), and 4M HCl in dioxane (1.6 mL). Yield: 1.174 g (56%). Mp: 88–92 °C. ^31^P{^1^H} NMR (161.98 MHz, CDCl_3_): *δ* 39.06 ppm. ^1^H NMR (400.13 MHz, CDCl_3_): *δ* 0.88 and 1.05 (both d, 3H + 3H, Me in *^i^*Pr, ^3^*J*_HH_ = 6.8 Hz), 2.36–2.44 (m, 1H, CH in *^i^*Pr), 2.81–2.88 and 2.96–3.05 (both m, 1H + 1H, CH_2_P(S)), 3.28 (br. s, 1H, CHN), 7.45–7.61 (m, 6H, *m*-H and *p*-H in P(S)Ph_2_), 7.81 (dd, 2H, *o*-H in P(S)Ph, ^3^*J*_HP_= 13.3 Hz, ^3^*J*_HH_ = 7.4 Hz), 7.92 (dd, 2H, *o*-H in P(S)Ph, ^3^*J*_HP_= 13.1 Hz, ^3^*J*_HH_ = 7.3 Hz), 8.81 (br. s, 3H, NH_3_^+^) ppm. ^13^C{^1^H} NMR (100.61 MHz, CDCl_3_): *δ* 17.92 and 18.14 (both s, Me in *^i^*Pr), 30.54 (d, CH_2_P(S), ^1^*J*_CP_ = 56.3 Hz), 30.64 (d, CH in *^i^*Pr, ^2^*J*_CP_ = 8.8 Hz), 54.10 (s, CHN), 128.97 (d, *m*-C in P(S)Ph, ^3^*J*_CP_ = 12.3 Hz), 129.09 (d, *m*-C in P(S)Ph, ^3^*J*_CP_ = 12.6 Hz), 129.48 (d, *ipso*-C in P(S)Ph, ^1^*J*_CP_ = 80.2 Hz), 130.89 (d, *o*-C in P(S)Ph, ^2^*J*_CP_ = 10.3 Hz), 131.79 (d, *o*-C in P(S)Ph, ^2^*J*_CP_ = 10.3 Hz), 132.07 (d, *ipso*-C in P(S)Ph, ^1^*J*_CP_ = 83.8 Hz), 132.17 (d, *p*-C in P(S)Ph, ^4^*J*_CP_ = 3.1 Hz), 132.38 (d, *p*-C in P(S)Ph, ^4^*J*_CP_ = 3.1 Hz) ppm. IR (*v*/cm^−1^, KBr): 513(m), 553(w), 581(vw), 612(m) and 626(m) (both *v*P=S), 692(s), 712(m), 745(m), 778(w), 811(w), 851(vw), 998(w), 1028(vw), 1071(w), 1103(s), 1160(w), 1184(w), 1228(vw), 1312(w), 1377(w), 1397(w), 1437(s), 1463(m), 1482(m), 1503(m), 1509(m), 1595(br, m), 2556(sh, m), 2648(sh, m), 2905(s), 2964(s), 3052(m). Anal. Calcd for C_17_H_23_ClNPS: C, 60.08; H, 6.82; N, 4.12. Found: C, 59.32; H, 6.85; N, 3.93%.

#### 3.2.2. Synthesis and Characteristics of Amide Ligands **2a**–**c**

**General procedure for the synthesis.** A stirred solution of picolinic acid (1.0 equiv.) in CH_2_Cl_2_ (10–13 mL) was cooled under an argon atmosphere to −5 °C using an ice/NaCl bath. Then, Et_3_N (ca. 1.5 equiv.) was added. The resulting mixture was stirred upon cooling for 30 min. Then, a solution of isobutyl chloroformate (1.1–1.3 equiv.) in CH_2_Cl_2_ (7–10 mL) was slowly added dropwise. The reaction mixture was stirred for 30 min, keeping the temperature below −5 °C. Then, a solution of the corresponding amine in situ generated from the hydrochloride salt **1a**–**c** (1.0 equiv.) and Et_3_N (1.6–2.0 equiv.) in CH_2_Cl_2_ (10 mL) was slowly added dropwise. During the addition, the temperature did not rise above 0 °C. The reaction mixture was stirred upon cooling for 30 min and, after removal of a cooling bath, for another 30 min and left overnight. The resulting mixture was diluted with CH_2_Cl_2_ and washed with water. The organic layer was separated, dried over anhydrous Na_2_SO_4_, and evaporated to dryness. The residue obtained was purified by column chromatography on silica gel (gradient elution with a petroleum ether–acetone mixture, from 5:1 to 3:1) to give the target ligands as white crystalline solids.

**(*S*)- and (*R*)-*N*-[2-(diphenylthiophosphoryl)-1-phenylethyl]picolinamides 2a**


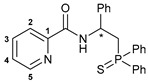


Compound **(*S*)-2a** was obtained according to the general procedure from picolinic acid (0.090 g, 0.731 mmol), isobutyl chloroformate (0.133 g, 0.974 mmol), amine precursor **(*S*)-1a** (0.273 g, 0.730 mmol), and Et_3_N (0.32 mL in total, 2.296 mmol). Yield: 0.193 g (60%). Mp: >59 °C (dec.). Compound **(*R*)-2a** was obtained according to the general procedure from picolinic acid (0.079 g, 0.642 mmol), isobutyl chloroformate (0.105 g, 0.769 mmol), amine precursor **(*R*)-1a** (0.240 g, 0.642 mmol), and Et_3_N (0.28 mL in total, 2.009 mmol). Yield: 0.207 g (73%). Mp: 67–72 °C. ^31^P{^1^H} NMR (121.49 MHz, CDCl_3_): *δ* 38.09 ppm. ^1^H NMR (300.13 MHz, CDCl_3_): *δ* 2.99–3.09 (m, 1H, CH_2_P(S)), 3.47–3.58 (m, 1H, CH_2_P(S)), 5.68–5.79 (m, 1H, CHN), 7.16–7.30 (m, 6H, H_Ar_), 7.35–7.48 (m, 6H, H_Ar_), 7.74–7.90 (m, 5H, *o*-H in P(S)Ph_2_ + H(C4) or H(C3)), 8.02 (d, 1H, H(C2), ^3^*J*_HH_ = 7.8 Hz), 8.41 (d, 1H, H(C5), ^3^*J*_HH_ = 4.6 Hz), 8.55 (br. d, 1H, NH, ^3^*J*_HH_ = 7.7 Hz) ppm. ^13^C{^1^H} NMR (100.61 MHz, CDCl_3_): *δ* 38.73 (d, CH_2_P(S), ^1^*J*_CP_ = 54.2 Hz), 50.02 (s, CHN), 122.07 and 126.02 (both s, C2 and C4), 126.75 (s, *m*-C or *o*-C in Ph), 127.61 (s, *p*-C in Ph), 128.42 (d, *m*-C in P(S)Ph, ^3^*J*_CP_ = 12.4 Hz), 128.62 (d, *m*-C in P(S)Ph, ^3^*J*_CP_ = 11.9 Hz), 128.64 (s, *o*-C or *m*-C in Ph), 130.97 (d, *o*-C in P(S)Ph, ^2^*J*_CP_ = 10.2 Hz), 131.08 (d, *p*-C in P(S)Ph, ^4^*J*_CP_ = 2.9 Hz), 131.24 (d, *o*-C in P(S)Ph, ^2^*J*_CP_ = 10.6 Hz), 131.43 (d, *p*-C in P(S)Ph, ^4^*J*_CP_ = 3.0 Hz), 132.22 (d, *ipso*-C in P(S)Ph, ^1^*J*_CP_ = 80.5 Hz), 132.88 (d, *ipso*-C in P(S)Ph, ^1^*J*_CP_ = 82.0 Hz), 137.03 (s, C3), 141.54 (d, *ipso*-C in Ph, ^3^*J*_CP_ = 9.8 Hz), 147.81 (s, C5), 149.46 (s, C1), 163.30 (s, C(O)NH) ppm. IR (*v*/cm^−1^, KBr): 485(w), 502(m), 527(w), 611(m), 621(m) and 629(m) (three *ν*P=S), 698(s), 741(s), 818(w), 998(m), 1103(m), 1158(vw), 1243(w), 1286(w), 1310(w), 1401(w), 1436(s), 1465(m), 1514(br, s) (C(O)NH), 1570(w), 1590(w), 1674(br, s) (*ν*C=O), 2927(vw), 3031(w), 3055(w), 3367(br, m) (*ν*NH). Anal. Calcd for C_26_H_23_N_2_OPS: C, 70.57; H, 5.24; N, 6.33. Found: C, 70.49; H, 5.36; N, 6.37 (**(*S*)-2a**); C, 70.51; H, 5.31; N, 6.38% (**(*R*)-2a**).

**(*S*)-*N*-[1-(Diphenylthiophosphoryl)-3-phenylpropan-2-yl]picolinamide (*S*)-2b**


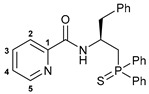


The compound was obtained according to the general procedure from picolinic acid (0.063 g, 0.512 mmol), isobutyl chloroformate (0.077 g, 0.564 mmol), amine precursor **(*S*)-1b** (0.199 g, 0.513 mmol), and Et_3_N (0.25 mL in total, 1.794 mmol). Yield: 0.128 g (55%). Mp: 59–64 °C. ^31^P{^1^H} NMR (121.49 MHz, CDCl_3_): *δ* 38.57 ppm. ^1^H NMR (300.13 MHz, CDCl_3_): *δ* 2.72–2.82 (m, 1H, CH_2_P(S)), 3.17–3.24 (m, 1H, CH_2_), 3.30–3.41 (m, 1H, CH_2_P(S) + 1H, CH_2_), 4.63–4.78 (m, 1H, CHN), 7.15–7.40 (m, 12H, H_Ar_), 7.68–7.87 (m, 5H, *o*-H in P(S)Ph_2_ + H(C4) or H(C3)), 7.94 (d, 1H, H(C2), ^3^*J*_HH_ = 7.8 Hz), 8.10 (br. d, 1H, NH, ^3^*J*_HH_ = 7.7 Hz), 8.34 (dd, 1H, H(C5), ^3^*J*_HH_ = 4.8 Hz, ^4^*J*_HH_ = 1.4) ppm. ^13^C{^1^H} NMR (100.61 MHz, CDCl_3_): *δ* 34.98 (d, CH_2_P(S), ^1^*J*_CP_ = 56.1 Hz), 40.89 (d, CH_2_, ^3^*J*_CP_ = 9.5 Hz), 48.52 (s, CHN), 121.70, 126.00 and 126.65 (three s, C2, C4 and *p*-C in Ph), 128.31 (d, *m*-C in P(S)Ph, ^3^*J*_CP_ = 12.4 Hz), 128.59 (s, *o*-C or *m*-C in Ph), 128.66 (d, *m*-C in P(S)Ph, ^3^*J*_CP_ = 13.1 Hz), 129.53 (s, *m*-C or *o*-C in Ph), 130.79 (d, *o*-C in P(S)Ph, ^2^*J*_CP_ = 10.1 Hz), 131.03 (d, *p*-C in P(S)Ph, ^4^*J*_CP_ = 3.1 Hz), 131.25 (d, *o*-C in P(S)Ph, ^2^*J*_CP_ = 10.7 Hz), 131.42 (d, *p*-C in P(S)Ph, ^4^*J*_CP_ = 3.0 Hz), 131.95 (d, *ipso*-C in P(S)Ph, ^1^*J*_CP_ = 80.0 Hz), 133.59 (d, *ipso*-C in P(S)Ph, ^1^*J*_CP_ = 81.4 Hz), 136.93 (s, C3), 137.94 (d, *ipso*-C in Ph), 147.78 (s, C5), 149.31 (s, C1), 163.82 (s, C(O)NH) ppm. IR (*v*/cm^−1^, KBr): 499(m), 536(vw), 612(m) and 624(m) (both *ν*P=S), 693(s), 742(s), 816(w), 998(m), 1042(vw), 1101(m), 1159(vw), 1244(w), 1286(w), 1399(w), 1435(s), 1464(m), 1519(br, s) (C(O)NH), 1570(m), 1590(m), 1671(br, s) (*ν*C=O), 2920(vw), 3055(w), 3358 (br, w) (*ν*NH). Anal. Calcd for C_27_H_25_N_2_OPS: C, 71.03; H, 5.52; N, 6.14. Found: C, 70.81; H, 5.54; N, 6.04%.

**(*S*)-*N*-[1-(Diphenylthiophosphoryl)-3-methylbutan-2-yl]picolinamide (*S*)-2c**


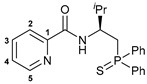


The compound was obtained according to the general procedure from picolinic acid (0.078 g, 0.634 mmol), isobutyl chloroformate (0.098 g, 0.718 mmol), amine precursor **(*S*)-1c** (0.215 g, 0.633 mmol), and Et_3_N (0.31 mL in total, 2.224 mmol). Yield: 0.202 g (78%). Mp: 95–99 °C. ^31^P{^1^H} NMR (121.49 MHz, CDCl_3_): *δ* 39.16 ppm. ^1^H NMR (300.13 MHz, CDCl_3_): *δ* 0.98 (d, 6H, Me in *^i^*Pr, ^3^*J*_HH_ = 6.7 Hz), 2.28–2.43 (m, 1H, CH in *i*Pr), 2.65–2.76 (m, 1H, CH_2_P(S)), 3.16–3.27 (m, 1H, CH_2_P(S)), 4.34–4.48 (m, 1H, CHN), 7.12–7.23 (m, 3H, *m*-H and *p*-H in P(S)Ph), 7.35 (ddd, 1H, H(C4), ^3^*J*_HH_ = 7.6 Hz, ^3^*J*_HH_ = 4.8 Hz, ^4^*J*_HH_ = 1.3 Hz), 7.38–7.45 (m, 3H, *m*-H and *p*-H in P(S)Ph), 7.72–7.78 (m, 1H, H(C3)), 7.80–7.97 (m, 6H, *o*-H in P(S)Ph_2_ + H(C2) + NH), 8.37 (dd, 1H, H(C5), ^3^*J*_HH_ = 4.8 Hz, ^4^*J*_HH_ = 1.7 Hz) ppm. ^13^C{^1^H} NMR (100.61 MHz, CDCl_3_): *δ* 18.14 and 19.48 (both s, Me in *^i^*Pr), 32.23 (d, CH in *^i^*Pr, ^3^*J*_CP_ = 9.9 Hz), 34.30 (d, CH_2_P(S), ^1^*J*_CP_ = 56.4 Hz), 51.26 (d, CHN, ^2^*J*_CP_ = 3.2 Hz), 121.89 and 125.80 (both s, C2 and C4), 128.19 (d, *m*-C in P(S)Ph, ^3^*J*_CP_ = 12.3 Hz), 128.63 (d, *m*-C in P(S)Ph, ^3^*J*_CP_ = 12.3 Hz), 130.76 (d, *o*-C in P(S)Ph, ^2^*J*_CP_ = 9.6 Hz), 130.82 (d, *p*-C in P(S)Ph, ^4^*J*_CP_ = 2.6 Hz), 131.32 (d, *p*-C in P(S)Ph, ^4^*J*_CP_ = 3.0 Hz), 131.36 (d, *o*-C in P(S)Ph, ^2^*J*_CP_ = 10.3 Hz), 131.69 (d, *ipso*-C in P(S)Ph, ^1^*J*_CP_ = 77.6 Hz), 134.04 (d, *ipso*-C in P(S)Ph, ^1^*J*_CP_ = 82.1 Hz), 136.90 (s, C3), 147.60 (s, C5), 149.45 (s, C1), 163.35 (s, C(O)NH) ppm. IR (*v*/cm^−1^, KBr): 495(w), 509(w), 570(vw), 614(m), 620(m) and 629(m) (three *ν*P=S), 689(w), 700(m), 705(m), 711(m), 741(w), 748(m), 755(w), 782(vw), 815(w), 878(vw), 997(w), 1043(vw), 1102(m), 1144(vw), 1180(vw), 1372(w), 1394(w), 1411(w), 1436(m), 1464(m), 1482(w), 1526(br, s) (C(O)NH), 1570(w), 1590(w), 1663(s) (*ν*C=O), 2872(vw), 2920(vw), 2961(w), 3055(w), 3369(br, w) (*ν*NH). Anal. Calcd for C_23_H_25_N_2_OPS: C, 67.63; H, 6.17; N, 6.86. Found: C, 67.61; H, 6.21; N, 6.94%.

#### 3.2.3. Synthesis and Characteristics of Pd(II) Pincer Complexes **3a**–**c**

**General method for the synthesis.** A solution of PdCl_2_(NCPh)_2_ (33 mg, 0.086 mmol) in CH_2_Cl_2_ (5 mL) was slowly added dropwise to a solution of the corresponding ligand (0.086 mmol) and Et_3_N (ca. 25 μL, 0.179 mmol) in CH_2_Cl_2_ (5 mL). The reaction mixture was left under ambient conditions for 1 day. The resulting mixture was purified by column chromatography on silica gel (eluent: first CH_2_Cl_2_ (to remove benzonitrile and the excess of Et_3_N), then CH_2_Cl_2_–EtOH (30:1 in the case of complexes **(*S*)-** and **(*R*)-3a**, **(*S*)-3b** or 100:1 in the case of complex **(*S*)-3c**)) to give the target products as light-orange (**(*S*)-** and **(*R*)-3a**, **(*S*)-3b**) or yellow (**(*S*)-3c**) crystalline solids.

**[κ^3^-*S*,*N*,*N*-(L)Pd(II)Cl] complexes (*S*)-3a and (*R*)-3a**


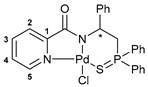


Yield: 44 mg (88%) (**(*S*)-3a**); 46 mg (92%) (**(*R*)-3a**). Mp: >269 °C (dec.) (**(*S*)-3a**); >217 °C (dec.) (**(*R*)-3a**). ^31^P{^1^H} NMR (121.49 MHz, CDCl_3_): *δ* 33.44 ppm. ^1^H NMR (300.13 MHz, CDCl_3_): *δ* 3.19–3.28 (m, 1H, CH_2_P(S)), 3.71–3.81 (m, 1H, CH_2_P(S)), 5.90–6.05 (m, 1H, CHN), 7.00–7.07 (m, 3H, H_Ar_), 7.23–7.35 (m, 4H, H_Ar_), 7.40–7.63 (m, 7H, H_Ar_), 7.89 (dd, 2H, *o*-H in P(S)Ph, ^3^*J*_HP_ =13.2 Hz, ^3^*J*_HH_ = 7.6 Hz), 7.95–8.01 (m, 2H, H_Ar_), 9.27 (d, 1H, H(C5), ^3^*J*_HH_ = 5.6 Hz) ppm. ^13^C{^1^H} NMR (100.61 MHz, CDCl_3_): *δ* 40.27 (d, CH_2_P(S), ^1^*J*_CP_ = 51.4 Hz), 50.59 (d, CHN, ^2^*J*_CP_ = 6.7 Hz), 125.57, 126.49 and 126.83 (three s, C2, C4, and *p*-C in Ph), 126.62 (d, *ipso*-C in P(S)Ph, ^1^*J*_CP_ = 81.0 Hz), 127.39 and 127.82 (both s, *m*-C and *o*-C in Ph), 128.75 (d, *m*-C in P(S)Ph, ^3^*J*_CP_ = 12.6 Hz), 129.41 (d, *m*-C in P(S)Ph, ^3^*J*_CP_ = 12.6 Hz), 130.07 (d, *ipso*-C in P(S)Ph, ^1^*J*_CP_ = 83.3 Hz), 131.49 (d, *o*-C in P(S)Ph_2_, ^2^*J*_CP_ =10.3 Hz), 132.31 (d, *p*-C in P(S)Ph, ^4^*J*_CP_ = 3.1 Hz), 132.85 (d, *p*-C in P(S)Ph, ^4^*J*_CP_ = 3.3 Hz), 139.28 (d, *ipso*-C in Ph, ^3^*J*_CP_ = 5.3 Hz), 139.76 (s, C3), 147.72 (s, C5), 154.37 (s, C1), 172.11 (s, C(O)N) ppm. IR (*v*/cm^−1^, KBr): 492(m), 558(w) and 585(w) (both *ν*P=S), 663(w), 691(m), 699(m), 731(w), 743(w), 755(m), 801(w), 840(w), 921(vw), 999(vw), 1061(w), 1103(m), 1191(vw), 1293(m), 1374(sh, m), 1383(m), 1438(m), 1451(w), 1480(w), 1494(w), 1570(w), 1597(s), 1620(s) (*ν*C=O), 2898(w), 2941(w), 3022(vw), 3055(w). Anal. Calcd for C_26_H_22_ClN_2_OPPdS: C, 53.53; H, 3.80; N, 4.80. Found: C, 53.64; H, 3.91; N, 4.81 (**(*S*)-3a**); C, 53.69; H, 4.01; N, 4.81% (**(*R*)-3a**).

**[κ^3^-*S*,*N*,*N*-(L)Pd(II)Cl] complex (*S*)-3b**


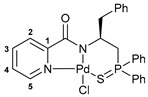


Yield: 44 mg (86%). Mp: >117 °C (dec.). ^31^P{^1^H} NMR (121.49 MHz, CDCl_3_): *δ* 33.00 ppm. ^1^H NMR (300.13 MHz, CDCl_3_): *δ* 2.91–3.01 (m, 1H, CH_2_P(S)), 3.06–3.20 (m, 2H, 1H, CH_2_P(S) + 1H, CH_2_Ph), 3.25–3.32 (m, 1H, CH_2_Ph), 5.13–5.30 (m, 1H, CHN), 7.11 (br. s, 5H, H_Ar_), 7.41–7.54 (m, 6H, H_Ar_), 7.58–7. 64 (m, 1H, H_Ar_), 7.70 (dd, 2H, *o*-H in P(S)Ph, ^3^*J*_HP_ = 13.1 Hz, ^3^*J*_HH_ = 8.1 Hz), 7.85–8.01 (m, 4H, H_Ar_), 9.16 (d, 1H, H(C5), ^3^*J*_HH_ = 5.2 Hz) ppm. ^13^C{^1^H} NMR (100.61 MHz, CDCl_3_): *δ* 34.58 (d, CH_2_P(S), ^1^*J*_CP_ = 52.0 Hz), 41.55 (d, CH_2_Ph, ^3^*J*_CP_ = 3.1 Hz), 50.66 (d, CHN, ^2^*J*_CP_ = 7.3 Hz), 125.35, 126.32 and 126.34 (three s, C2, C4, and *p*-C in Ph), 128.23 (d, *ipso*-C in P(S)Ph, ^1^*J*_CP_ = 80.8 Hz), 128.35 (s, *m*-C or *o*-C in Ph), 129.37 (d, *m*-C in P(S)Ph, ^3^*J*_CP_ = 12.9 Hz), 129.40 (d, *m*-C in P(S)Ph, ^3^*J*_CP_ = 12.4 Hz), 129.50 (s, *o*-C or *m*-C in Ph), 131.34 (d, *o*-C in P(S)Ph, ^2^*J*_CP_ =10.1 Hz), 131.38 (d, *ipso*-C in P(S)Ph, ^1^*J*_CP_ = 83.2 Hz), 131.53 (d, *o*-C in P(S)Ph, ^2^*J*_CP_ =10.4 Hz), 132.71 (d, *p*-C in P(S)Ph, ^4^*J*_CP_ = 3.1 Hz), 133.03 (d, *p*-C in P(S)Ph, ^4^*J*_CP_ = 3.0 Hz), 138.30 (s, *ipso*-C in Ph), 139.60 (s, C3), 147.69 (s, C5), 154.73 (s, C1), 171.78 (s, C(O)N) ppm. IR (*v*/cm^−1^, KBr): 492(w), 521(vw), 571(w) and 581(w) (both *ν*P=S), 687(m), 702(m), 728(w), 738(w), 753(w), 764(sh, w), 811(w), 998(vw), 1031(vw), 1049(w), 1104(m), 1160(vw), 1187(vw), 1290(m), 1379(br, m), 1436(m), 1481(w), 1494(w), 1569(m), 1597(s), 1617(s) (*ν*C=O), 2849(vw), 2911(vw), 2971(vw), 3026(w), 3056(w). Anal. Calcd for C_27_H_24_ClN_2_OPPdS: C, 54.28; H, 4.05; N, 4.69. Found: C, 54.44; H, 4.31; N, 4.61%.

**[κ^3^-*S*,*N*,*N*-(L)Pd(II)Cl] complex (*S*)-3c**


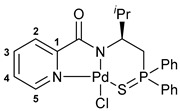


Yield: 37 mg (79%). Mp: >225 °C (dec.). ^31^P{^1^H} NMR (121.49 MHz, CDCl_3_): *δ* 33.24 ppm. ^1^H NMR (300.13 MHz, CDCl_3_): *δ* 0.71 (d, 3H, Me in *^i^*Pr, ^3^*J*_HH_ = 6.6 Hz), 1.01 (d, 3H, Me in *^i^*Pr, ^3^*J*_HH_ = 6.6 Hz), 2.54–2.66 (m, 1H, CH in *^i^*Pr), 2.98–3.08 (m, 1H, CH_2_P(S)), 3.32–3.42 (m, 1H, CH_2_P(S)), 4.40–4.59 (m, 1H, CHN), 7.43–7.47 (m, 1H, H_Ar_), 7.51–7.67 (m, 6H, H_Ar_), 7.82–7.90 (m, 2H, *o*-H in P(S)Ph), 7.93–8.03 (m, 4H, H_Ar_), 9.18 (d, 1H, H(C5), ^3^*J*_HH_ = 5.5 Hz) ppm. ^13^C{^1^H} NMR (100.61 MHz, CDCl_3_): *δ* 19.70 and 20.09 (both s, Me in *^i^*Pr), 31.76 (d, CH in *^i^*Pr, ^3^*J*_CP_ = 4.0 Hz), 35.03 (d, CH_2_P(S), ^1^*J*_CP_ = 51.2 Hz), 55.14 (d, CHN, ^2^*J*_CP_ = 6.8 Hz), 125.59 and 126.31 (both s, C2 and C4), 128.46 (d, *ipso*-C in P(S)Ph, ^1^*J*_CP_ = 79.9 Hz), 129.40 (d, *m*-C in P(S)Ph, ^3^*J*_CP_ = 12.8 Hz), 129.42 (d, *m*-C in P(S)Ph, ^3^*J*_CP_ = 12.5 Hz), 131.29 (d, *ipso*-C in P(S)Ph, ^1^*J*_CP_ = 83.1 Hz), 131.35 (d, *o*-C in P(S)Ph, ^2^*J*_CP_ = 10.1 Hz), 131.51 (d, *o*-C in P(S)Ph, ^2^*J*_CP_ = 10.3 Hz), 132.73 (d, *p*-C in P(S)Ph, ^4^*J*_CP_ = 3.0 Hz), 133.04 (d, *p*-C in P(S)Ph, ^4^*J*_CP_ = 3.1 Hz), 139.55 (s, C3), 147.66 (s, C5), 154.82 (s, C1), 172.14 (s, C(O)N) ppm. IR (*v*/cm^−1^, KBr): 481(w), 494(w), 503(w), 549(w), 587(m) (*ν*P=S), 641(vw), 689(m), 712(m), 728(w), 746 (sh, w), 756(m), 806(w), 835(w), 998(w), 1053(w), 1103(m), 1139(w), 1184(vw), 1293(m), 1373(br, m), 1391(sh, m), 1438(m), 1479(w), 1569(m), 1595(br, s), 1622(br, s) (*ν*C=O), 2870(vw), 2930(w), 2965(w), 3076(vw). Anal. Calcd for C_23_H_24_ClN_2_OPPdS: C, 50.29; H, 4.40; N, 5.10. Found: C, 49.98; H, 4.58; N, 5.07%.

#### 3.2.4. Synthesis and Characteristics of Compound **6**

***N*-[2-(Diphenylthiophosphoryl)-2-phenylethyl]picolinamide 6**


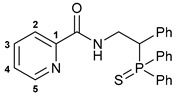


A stirred solution of picolinic acid (0.049 g, 0.398 mmol) in CH_2_Cl_2_ (10 mL) was cooled under an argon atmosphere to −5 °C using an ice/NaCl bath. Then, Et_3_N (0.10 mL, 0.717 mmol) was added. The resulting mixture was stirred upon cooling for 20 min. Then, a solution of isobutyl chloroformate (0.067 g, 0.491 mmol) in CH_2_Cl_2_ (5 mL) was slowly added dropwise. The reaction mixture was stirred for 35 min, keeping the temperature below –5 °C. Then, a solution of amine **5** (0.134 g, 0.397 mmol) in CH_2_Cl_2_ (15 mL) was slowly added dropwise. During the addition, the temperature did not rise above 0 °C. The reaction mixture was stirred upon cooling for 30 min and, after removal of a cooling bath, for another 30 min and left overnight. The resulting mixture was diluted with CH_2_Cl_2_ and washed with water. The organic layer was separated, dried over anhydrous Na_2_SO_4_, and evaporated to dryness. The residue obtained was purified by column chromatography on silica gel (gradient elution with a petroleum ether–acetone mixture, from 5:1 to 4:1) to give the target ligand as a white crystalline solid. Yield: 0.103 g (59%). Mp: >66 °C (dec.). ^31^P{^1^H} NMR (121.49 MHz, CDCl_3_): *δ* 47.31 ppm. ^1^H NMR (300.13 MHz, CDCl_3_): *δ* 4.01–4.15 and 4.21–4.33 (both m, 1H + 1H, CH_2_N), 4.58–4.66 (m, 1H, CHP(S)), 7.09–7.18 (m, 5H, H_Ar_), 7.22–7.47 (m, 9H, H_Ar_), 7.75 (dt, 1H, H(C3), ^3^*J*_HH_ = 7.7 Hz, ^4^*J*_HH_ = 1.7 Hz), 8.03 (d, 1H, H(C2), ^3^*J*_HH_ = 7.7 Hz), 8.08 (vt, 1H, NH, ^3^*J*_HH_ = 6.1 Hz), 8.27 (dd, 2H, *o*-H in P(S)Ph, ^3^*J*_HP_ = 12.3 Hz, ^3^*J*_HH_ = 7.8 Hz), 8.33 (d, 1H, H(C5), ^3^*J*_HH_ = 4.7 Hz) ppm. ^13^C{^1^H} NMR (100.61 MHz, CDCl_3_): *δ* 41.13 (d, CH_2_N, ^2^*J*_CP_ = 4.6 Hz), 44.92 (d, CHP(S), ^1^*J*_CP_ = 50.7 Hz), 121.74 and 126.09 (both s, C2 and C4), 127.63 (d, *p*-C in Ph, ^5^*J*_CP_ = 3.0 Hz), 127.87 (d, *m*-C in P(S)Ph, ^3^*J*_CP_ = 12.1 Hz), 128.12 (d, *m*-C in Ph, ^4^*J*_CP_ = 2.3 Hz), 128.66 (d, *m*-C in P(S)Ph, ^3^*J*_CP_ = 11.9 Hz), 129.78 (d, *o*-C in Ph, ^3^*J*_CP_ = 5.9 Hz), 131.00 (d, *p*-C in P(S)Ph, ^4^*J*_CP_ = 3.0 Hz), 131.22 (d, *ipso*-C in P(S)Ph, ^1^*J*_CP_ = 76.4 Hz), 131.39 (d, *o*-C in P(S)Ph, ^2^*J*_CP_ = 9.7 Hz), 131.42 (d, *p*-C in P(S)Ph, ^4^*J*_CP_ = 2.9 Hz), 131.57 (d, *ipso*-C in P(S)Ph, ^1^*J*_CP_ = 81.9 Hz), 131.78 (d, *o*-C in P(S)Ph, ^2^*J*_CP_ = 9.7 Hz), 133.65 (d, *ipso*-C in Ph, ^2^*J*_CP_ = 4.7 Hz), 137.01 (s, C3), 147.99 (s, C5), 149.18 (s, C1), 165.02 (s, C(O)NH) ppm. IR (*v*/cm^−1^, KBr): 484(w), 532(m), 600(w), 613(m) and 630(m) (three *ν*P=S), 697(s), 710(sh, m), 749(m), 811(w), 998(w), 1041(vw), 1101(m), 1160(w), 1245(w), 1311(w), 1361(w), 1436(s), 1453(m), 1465(m), 1494(m), 1521(br, s) (C(O)NH), 1570(w), 1591(w), 1672(br, s) (*ν*C=O), 2854(vw), 2925(w), 3056(w), 3383(br, w) (*ν*NH). Anal. Calcd for C_26_H_23_N_2_OPS: C, 70.57; H, 5.24; N, 6.33. Found: C, 70.34; H, 5.25; N, 6.44%.

#### 3.2.5. Synthesis and Characteristics of Compound **7**

**[κ^3^-*S*,*N*,*N*-(L)Pd(II)Cl] complex 7**


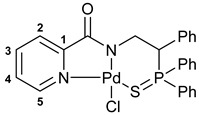


A solution of PdCl_2_(NCPh)_2_ (27 mg, 0.070 mmol) in CH_2_Cl_2_ (3 mL) was slowly added dropwise to a solution of ligand **6** (31 mg, 0.070 mmol) and Et_3_N (15 μL, 0.108 mmol) in CH_2_Cl_2_ (5 mL). The reaction mixture was left under ambient conditions for 1 day. The resulting mixture was purified by column chromatography on silica gel (eluent: first CH_2_Cl_2_ (to remove benzonitrile and the excess of Et_3_N), then CH_2_Cl_2_–EtOH (100:1)) to give the target product as a light-orange crystalline solid. Yield: 29 mg (71%). Mp: >247 °C (dec.). ^31^P{^1^H} NMR (121.49 MHz, CDCl_3_): *δ* 49.22 ppm. ^1^H NMR (300.13 MHz, CDCl_3_): *δ* 3.78–3.92 (m, 1H, CH_2_N), 4.30–4.37 (m, 1H, CHP(S)), 4.50–4.66 (m, 1H, CH_2_N), 7.01–7.14 (m, 5H, H_Ar_), 7.20–7.26 (m, 2H, *m*-H in P(S)Ph), 7.38–7.47 (m, 4H, H_Ar_), 7.54–7.65 (m, 3H, H_Ar_), 7.89–7.98 (m, 2H, H_Ar_), 8.09–8.17 (m, 2H, *o*-H in P(S)Ph), 9.09 (d, 1H, H(C5), ^3^*J*_HH_ = 5.3 Hz) ppm. ^13^C{^1^H} NMR (100.61 MHz, CDCl_3_): *δ* 46.74 (d, CH_2_N, ^2^*J*_CP_ = 3.8 Hz), 49.95 (d, CHP(S), ^1^*J*_CP_ = 44.0 Hz), 124.67 (d, *ipso*-C in P(S)Ph, ^1^*J*_CP_ = 80.5 Hz), 125.25 and 126.43 (both s, C2 and C4), 128.02 (d, *p*-C in Ph, ^5^*J*_CP_ = 3.6 Hz), 128.26 (d, *m*-C in P(S)Ph, ^3^*J*_CP_ = 12.2 Hz), 128.66 (d, *m*-C in Ph, ^4^*J*_CP_ = 2.8 Hz), 129.06 (d, *o*-C in Ph, ^3^*J*_CP_ = 6.0 Hz), 129.43 (d, *m*-C in P(S)Ph, ^3^*J*_CP_ = 12.4 Hz), 130.12 (d, *ipso*-C in P(S)Ph, ^1^*J*_CP_ = 79.9 Hz), 132.39 (d, *o*-C in P(S)Ph, ^2^*J*_CP_ = 9.6 Hz), 132.63 (d, *p*-C in P(S)Ph, ^4^*J*_CP_ = 3.0 Hz), 132.91 (d, *o*-C in P(S)Ph, ^2^*J*_CP_ = 9.3 Hz), 133.09 (d, *p*-C in P(S)Ph, ^4^*J*_CP_ = 3.0 Hz), 134.17 (d, *ipso*-C in Ph, ^2^*J*_CP_ = 5.2 Hz), 139.62 (s, C3), 147.72 (s, C5), 154.08 (s, C1), 172.02 (s, C=O) ppm. IR (*v*/cm^−1^, KBr): 489(vw), 509(w), 518(m), 538(vw), 590(m) (*ν*P=S), 632(vw), 687(m), 700(w), 752(m), 767(w), 815(vw), 983(w), 998(vw), 1031(vw), 1048(vw), 1101(m), 1157(w), 1189(vw), 1259(vw), 1288(w), 1351(w), 1392(m), 1436(m), 1452(w), 1482(w), 1492(w), 1569(m), 1594(s), 1619(s) (*ν*C=O), 2855(vw), 2925(vw), 3026(w), 3056(w). Anal. Calcd for C_26_H_22_ClN_2_OPPdS: C, 53.53; H, 3.80; N, 4.80. Found: C, 53.24; H, 3.91; N, 4.85%.

### 3.3. X-Ray Crystallography

Single crystals of compounds **(*S*)-3a**, **(*R*)-3a** and **(*S*)-3c** were grown by the double-layer crystallization technique (CH_2_Cl_2_–Et_2_O, 1:2). X-ray diffraction data were collected at 100 K with a Bruker APEXII Quazar CCD diffractometer (Bruker AXS GmbH, Karlsruhe, Germany), using graphite monochromated Mo-Kα radiation (λ = 0.71073 Å). Using Olex2 v. 1.5 [52], the structures were solved with the ShelXT 2018/2 [53] structure solution program using intrinsic phasing and refined with the XL 2008 [54] refinement package using Least Squares minimization against F^2^_hkl_ in anisotropic approximation for non-hydrogen atoms. Positions of hydrogen atoms were calculated, and they were refined in the isotropic approximation within the riding model. Crystal data and structure refinement parameters are given in Table 4. CCDC 2535001 (**(*S*)-3a**), 2535003 (**(*R*)-3a**), and 2535002 (**(*S*)-3c**) contain the supplementary crystallographic data for this paper.

### 3.4. Cytotoxicity Assay

The cytotoxic activity of the ligands and complexes obtained (enantiomerically pure compounds **2a**–**c** and **3a**–**c** and racemic derivatives **6** and **7**) was investigated on human colorectal carcinoma (HCT116), breast cancer (MCF7), prostate adenocarcinoma (PC3), chronic myelogenous leukemia (K562 and K562/iS9), multiple plasmacytoma (AMO1), and acute lymphoblastic leukemia (MOLT4) cell lines, as well as human embryonic kidney (HEK293) and mammary epithelial (HBL100 and HBL100/Dox) cells used as non-cancerous cell lineages. All the cell lines were obtained from American Type Culture Collection (ATCC) (Manassas, VA, USA). The tested compounds are not soluble in water and were initially dissolved in DMSO. Cisplatin was obtained from a commercial source (as an infusion concentrate in natural saline solution). The experiments were performed in triplicate using the conventional MTT assay (ICN Biomedicals, Eschwege, Germany) according to the previously published procedure [40].

### 3.5. Apoptosis-Induction Studies

The apoptosis inducing ability of complexes **(*S*)-3a** and **(*R*)-3a** was investigated on K562 and K562/iS9 cells, cultured in the medium containing 10 μM of the tested palladocycle for 20 h. The experiments were performed following the published procedure [40]. The analysis was carried out on a FACScan flow cytometer (Becton Dickinson, Franklin Lakes, NJ, USA) using the CellQuest software (version 3.3).

### 3.6. Cell Cycle Progression

Chronic myelogenous leukemia cells K562/iS9 were seeded into 12-well plates (3 × 10^5^ cells per well) in complete culture medium. Briefly, the cells were treated with complexes **(*S*)-3a** and **(*R*)-3a** (15 μM) and incubated for 24, 48 and 72 h. After incubation, the cells were harvested and washed once with phosphate-buffered saline (PBS). The pellets were then lysed in the solution containing 0.1% sodium citrate, 0.3% NP-40, 100 μg/mL RNAse A, and 10 μg/mL PI. The cells were analyzed using a BD FACS Canto II flow cytometer in a PerCP-Cy5.5 channel (filter 695/40) (Becton Dickinson, Franklin Lakes, NJ, USA). The fluorescence emitted from the PI–DNA complex was exited at 488 nm using at least 10,000 cells per sample. FACSDiva software, version 8.0 (BD Biosci., Franklin Lakes, NJ, USA) was used to determine the percentage of cells in the G0/G1, S, and G2/M phases, as well as dead cells with degraded DNA (subG1).

### 3.7. Western Blotting and Immunocytochemical Staining

For Western blot analysis, PC3 prostate cancer cells were scraped and lysed using RIPA buffer (ThermoScientific, Waltham, MA, USA). The following procedures are described in detail in Ref. [55]. Briefly, after SDS-PAGE electrophoresis and blotting onto a nitrocellulose membrane, blots were incubated at 4 °C overnight with specific primary antibodies (see Table S1 in the Supporting Information). After that, the blots were washed with TBS/Tween-20 and incubated at room temperature for 1 h with horseradish peroxidase (HRP)-conjugated secondary antibodies (Jackson ImmunoResearch, West Grove, PA, USA). The membranes were washed with TBST and protein bands were visualized with enhanced chemiluminescence (ECL) substrate (ThermoScientific, Waltham, MA, USA) and imaged by Image Quant LAS4000 (GE HealthCare, Chicago, IL, USA). The β-Actin protein level served as a loading control. ImageJ software, version 2.14.0 (National Institutes of Health, Bethesda, MD, USA), was applied to measure the expression of investigated proteins.

For immunocytochemical staining, PC3 prostate cancer cells cultured on coverslips were treated with complexes **(*S*)-3a** or **(*R*)-3a** (22 μM), cisplatin or left untreated for 24 h. The fixation, permeabilization, and incubation conditions with primary and secondary antibodies (Alexa Fluor^™^ 488 goat anti-rabbit, ThermoScientific, USA) as well as immunofluorescent visualization procedures were described previously [56]. The cell nuclei were stained with Hoechst 33285 (Sigma-Aldrich, Saint Louis, MO, USA).

### 3.8. Antibacterial Activity Studies

The microbial strains *Bacillus subtillis* (VKM B-501^T^), *Micrococcus luteus* (VKM Ac-2230^T^), *Escherichia coli* (VKM B-3674), and *Groenewaldozyma auringiensis* (VKM Y-2927) were used for antimicrobial activity tests. All strains were obtained from the All-Russian Collection of Microorganisms (VKM) at the Skryabin Institute of Biochemistry and Physiology of Microorganisms, Pushchino Scientific Center of Biological Research. The experiments were performed according to the published methodology [38]. All strains were grown on the following medium (g/L): aminopeptide 60 mL; tryptone 5.0 g; yeast extract 1.0 g; soybean extract 30 mL; bacto-agar 15.0 g; final pH 7.2. *G. auringiensis* was grown on malt extract medium containing the following medium (g/L): malt extract 12.75; dextrin 2.75; glycerol 2.35; gelatin peptone 0.78; bacto-agar 15; final pH 5.4.

## 4. Conclusions

A series of chiral β-(aminoalkyl)phosphine sulfides with a stereogenic carbon center were synthesized and used as the precursors for new functionalized amide ligands, which were successfully coordinated to Pd(II) ions in a tridentate monoanionic fashion. The resulting *S*,*N*,*N*-pincer complexes featuring a robust deprotonated amide core exhibited potent cytotoxic activity against several solid and hematopoietic human cancer cell lines, with the marked selectivity towards blood cancer cells. The latter along with the reduced effect on non-cancerous cells comprised important advantages of the palladocycles obtained in this work over their α-thiophosphorylated analogs and the clinically used drug—cisplatin. A closer inspection of some bioactivity aspects revealed high apoptosis-inducing ability of the β-thiophosphorylated picolinamide-based Pd(II) complexes, with a comparable level of efficiency on parental and doxorubicin-resistant cell lines. Furthermore, these compounds may cause serious DNA damage through double-strand breaks, without activation of the repair system. In addition, one of the phenyl-tethered derivatives was shown to possess moderate antibacterial activity against several Gram-positive and Gram-negative strains. Thus, the thiophosphoryl-substituted carboxamides represent a perspective platform for the development of novel metal-based therapeutics.

## Figures and Tables

**Table 1:** Cytotoxicities of the complexes obtained against selected human solid cancer and non-cancerous cell lines.

Entry	Compound	IC_50_ ± SD ^1^, μM					
		HCT116	MCF7	PC3	HEK293	HBL100	HBL100/Dox
**1**	**(*S*)-3a**	22.0 ± 2.5	45.3 ± 3.8	22.8 ± 4.4	34.5 ± 2.5	34.0 ± 1.0	32.5 ± 2.5
**2**	**(*R*)-3a**	21.0 ± 3.5	45.3 ± 3.7	24.0 ± 1.6	32.7 ± 3.8	32.3 ± 3.8	29.0 ± 2.0
**3**	**(*S*)-3b**	21.0 ± 2.5	46.5 ± 1.5	26.0 ± 1.5	27.0 ± 3.4	30.0 ± 4.5	35.0 ± 3.0
**4**	**(*S*)-3c**	20.7 ± 2.1	44.0 ± 4.2	20.5 ± 2.0	24.7 ± 2.4	32.0 ± 2.0	30.0 ± 5.0
**5**	**7**	32.7 ± 3.9	54.0 ± 5.0	19.7 ± 0.4	18.3 ± 2.6	47.3 ± 2.5	44.0 ± 4.6
**6**	Cisplatin	18.0 ± 2.0	25.0 ± 4.0	16.0 ± 3.0	12.5 ± 1.5	14.6 ± 3.6	23.6 ± 3.6

^1^ SD is the standard deviation of the value.

**Table 2:** Cytotoxicities of the complexes obtained against selected human hematopoietic cancer cell lines.

Entry	Compound	IC_50_ ± SD ^1^, μM			
		K562	K562/iS9	AMO1	MOLT4
**1**	**(*S*)-3a**	9.5 ± 1.0	5.5 ± 0.8	4.8 ± 1.4	9.7 ± 1.8
**2**	**(*R*)-3a**	7.8 ± 1.2	5.7 ± 1.7	3.6 ± 0.4	7.0 ± 2.0
**3**	**(*S*)-3b**	6.1 ± 0.3	3.4 ± 0.2	4.1 ± 0.9	8.8 ± 0.2
**4**	**(*S*)-3c**	6.3 ± 1.3	5.5 ± 0.5	5.6 ± 0.4	11.2 ± 0.8
**5**	**7**	7.7 ± 1.0	7.6 ± 0.9	5.6 ± 0.6	8.2 ± 0.3
**6**	Cisplatin	15.5 ± 0.5	16.0 ± 2.0	3.2 ± 0.6	6.5 ± 1.0

^1^ SD is the standard deviation of the value.

**Table 3:** Antibacterial activity of complex **(*R*)-3a**.

Compound, Concentration		Inhibition Zone, mm			
		*B. subtillis* (VKM B-501^T^)	*M. luteus* (VKM Ac-2230^T^)	*E. coli* (VKM B-3674)	*G. auringiensis* (VKM Y-2927)
Complex **(*R*)-3a**	0.25 mM	0	0.5 ± 0.3	0	0
	2.50 mM	5.0 ± 0.8	5.5 ± 0.8	3.0 ± 0.6	0
DMSO, 15%		0	0	0	0
Km, 50 mg/mL		10.0 ± 0.8	15.0 ± 1.0	0	0

**Table 4:** Crystal data and structure refinement parameters for compounds **(*S*)-3a**, **(*R*)-3a** and **(*S*)-3c**.

	(*S*)-3a	(*R*)-3a	(*S*)-3c
Empirical formula	C_26_H_22_ClN_2_OPPdS	C_26_H_22_ClN_2_OPPdS	C_23_H_24_ClN_2_OPPdS
Formula weight	583.33	583.33	549.32
T, K	100	100	100
Crystal system	Orthorhombic	Orthorhombic	Tetragonal
Space group	*P2_1_2_1_2_1_*	*P2_1_2_1_2_1_*	*P4_3_2_1_2*
Z	4	4	8
a, Å	9.6236(14)	9.6019(3)	8.6445(11)
b, Å	14.317(2)	14.3092(5)	8.6445(11)
c, Å	17.625(3)	17.6009(6)	61.221(11)
α, °	90	90	90
β, °	90	90	90
γ, °	90	90	90
V, Å^3^	2428.4(6)	2418.28(14)	4574.9(14)
D_calc_ (g cm^−1^)	1.596	1.602	1.595
Linear absorption, μ (cm^−1^)	10.48	10.53	11.07
F(000)	1176	1176	2224
2θmax, °	58	58	52
Reflections measured	29795	29935	34446
Independent reflections	6444	6419	4499
Observed reflections [I > 2σ(I)]	5330	5430	4091
Parameters	300	299	273
R1	0.0356	0.0349	0.0556
wR2	0.0625	0.0665	0.1023
GOF	0.973	0.996	1.106
Δρ_max_/Δρ_min_ (e Å^−3^)	0.461/−0.456	0.358/−0.540	0.634/−0.721
CCDC	2535001	2535003	2535002

## Data Availability

The original contributions presented in this study are included in the article/Supplementary Materials. Further inquiries can be directed to the corresponding author.

## Supplementary Materials

The following supporting information can be downloaded at https://www.mdpi.com/article/10.3390/ijms27083525/s1.
