# Supplementary material for: Picolinamides with β-Thiophosphorylated Amine Residues as a Useful Scaffold to Generate Biologically Active Pd(II) Pincer Complexes
Source: Int J Mol Sci. 2026 Apr 15;27(8):3525. doi: 10.3390/ijms27083525 (PMC13115956; doi:10.3390/ijms27083525)
Supplement: Supplementary file 1 [file ijms-27-03525-s001.zip › ijms-4218005-supplementary.pdf]

Supporting information

Picolinamides with β-thiophosphorylated amine residues as a useful scaffold to generate biologically active Pd(II) pincer complexes

Diana V. Aleksanyan [1,*], Aleksandra A. Kalashnikova [1], Anna Yu. Katranova [1,2], Ekaterina Yu. Rybalkina [3], Nikolay N. Kalitin [3], Yulia L. Volodina [3], Yana V. Ryzhmanova [4], Yulia V. Nelyubina [5], Oleg I. Artyushin [1], Zinaida S. Klemenkova [1] and Vladimir A. Kozlov [1]

[1] A. N. Nesmeyanov Institute of Organoelement Compounds, Russian Academy of Sciences, ul. Vavilova 28, Str. 1, Moscow 119334, Russia

[2] Faculty of Chemical and Pharmaceutical Technologies and Biomedical Products, D. I. Mendeleev University of Chemical Technology of Russia, Miusskaya pl. 9, Str. 1, Moscow 125047, Russia

[3] N. N. Blokhin National Medical Research Center of Oncology of the Ministry of Health of the Russian Federation, Kashirskoe Shosse 23, Moscow 115478, Russia

[4] Institute of Biochemistry and Physiology of Microorganisms, Federal Research Center "Pushchino Scientific Center of Biological Research of the Russian Academy of Sciences", pr. Nauki 5, Pushchino 142292, Russia

[5] Advanced Engineering School, ITMO University, ul. Lomonosova 9, St. Petersburg 191002, Russia

* Correspondence: aleksanyan.diana@ineos.ac.ru

Table of contents

	Page
**Figure S1.** $^{31}P\{^1H\}$ NMR spectrum of amine precursor **1a** (121.49 MHz, $CDCl_3$)	S2
**Figure S2.** ^1H NMR spectrum of amine precursor **1a** (300.13 MHz, $CDCl_3$)	S3
**Figure S3.** $^{13}C\{^1H\}$ NMR spectrum of amine precursor **1a** (100.61 MHz, $CDCl_3$)	S4
**Figure S4.** $^{31}P\{^1H\}$ NMR spectrum of ligand **2a** (121.49 MHz, $CDCl_3$)	S5
**Figure S5.** ^1H NMR spectrum of ligand **2a** (300.13 MHz, $CDCl_3$)	S6
**Figure S6.** $^{13}C\{^1H\}$ NMR spectrum of ligand **2a** (100.61 MHz, $CDCl_3$)	S7
**Figure S7.** $^{31}P\{^1H\}$ NMR spectrum of complex **3a** (121.49 MHz, $CDCl_3$)	S8
**Figure S8.** ^1H NMR spectrum of complex **3a** (300.13 MHz, $CDCl_3$)	S9
**Figure S9.** $^{13}C\{^1H\}$ NMR spectrum of complex **3a** (100.61 MHz, $CDCl_3$)	S10
**Figure S10.** $^{31}P\{^1H\}$ NMR spectrum of ligand **6** (121.49 MHz, $CDCl_3$)	S11
**Figure S11.** ^1H NMR spectrum of ligand **6** (300.13 MHz, $CDCl_3$)	S12
**Figure S12.** $^{13}C\{^1H\}$ NMR spectrum of ligand **6** (100.61 MHz, $CDCl_3$)	S13
**Figure S13.** $^{31}P\{^1H\}$ NMR spectrum of complex **7** (121.49 MHz, $CDCl_3$)	S14
**Figure S14.** ^1H NMR spectrum of complex **7** (300.13 MHz, $CDCl_3$)	S15
**Figure S15.** $^{13}C\{^1H\}$ NMR spectrum of complex **7** (100.61 MHz, $CDCl_3$)	S16
**Table S1.** Primary antibodies used in the western blot analyses	S17

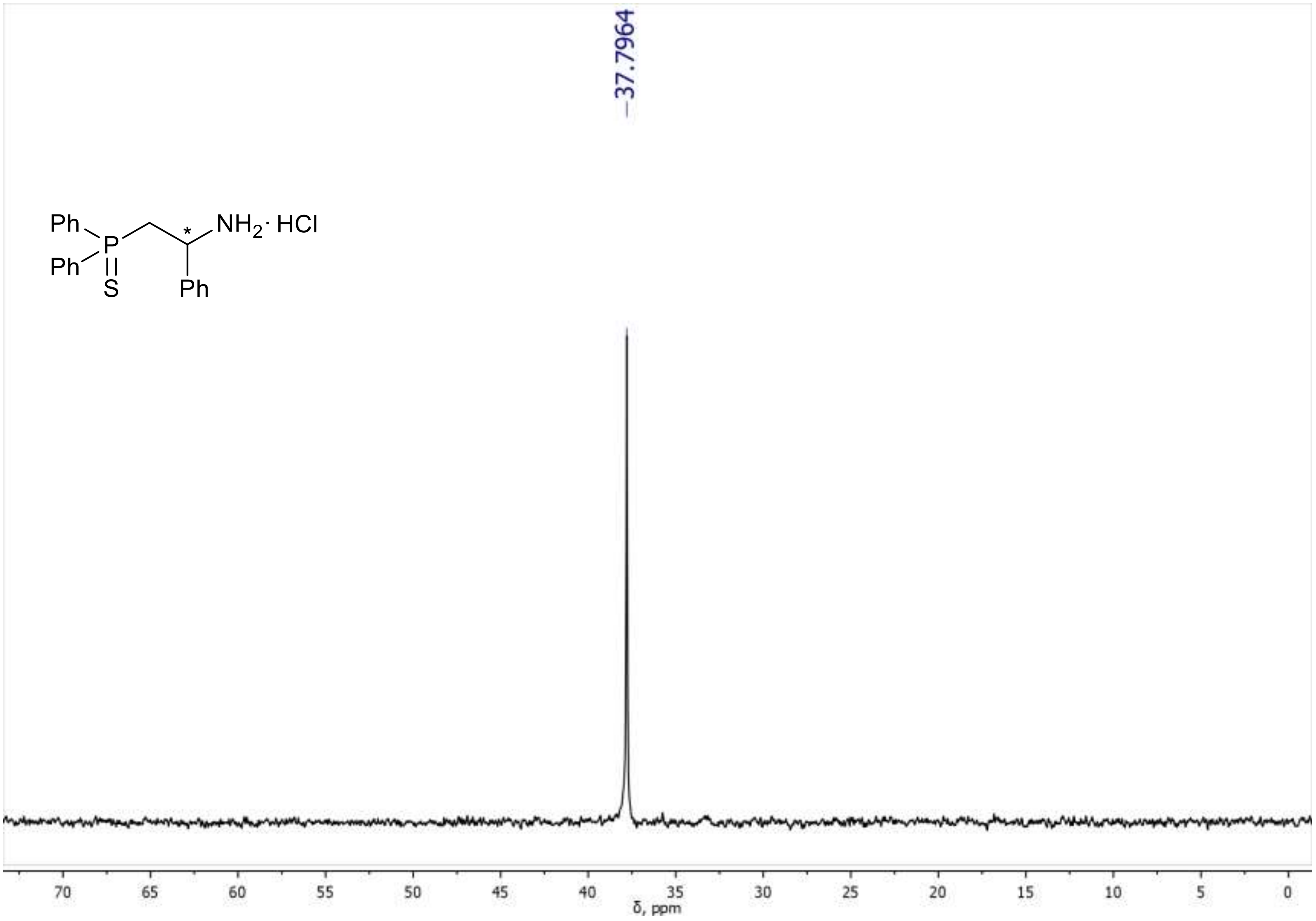

Figure S1. $^{31}P\{^{1}H\}$ NMR spectrum of amine precursor **1a** (121.49 MHz, $CDCl_3$)

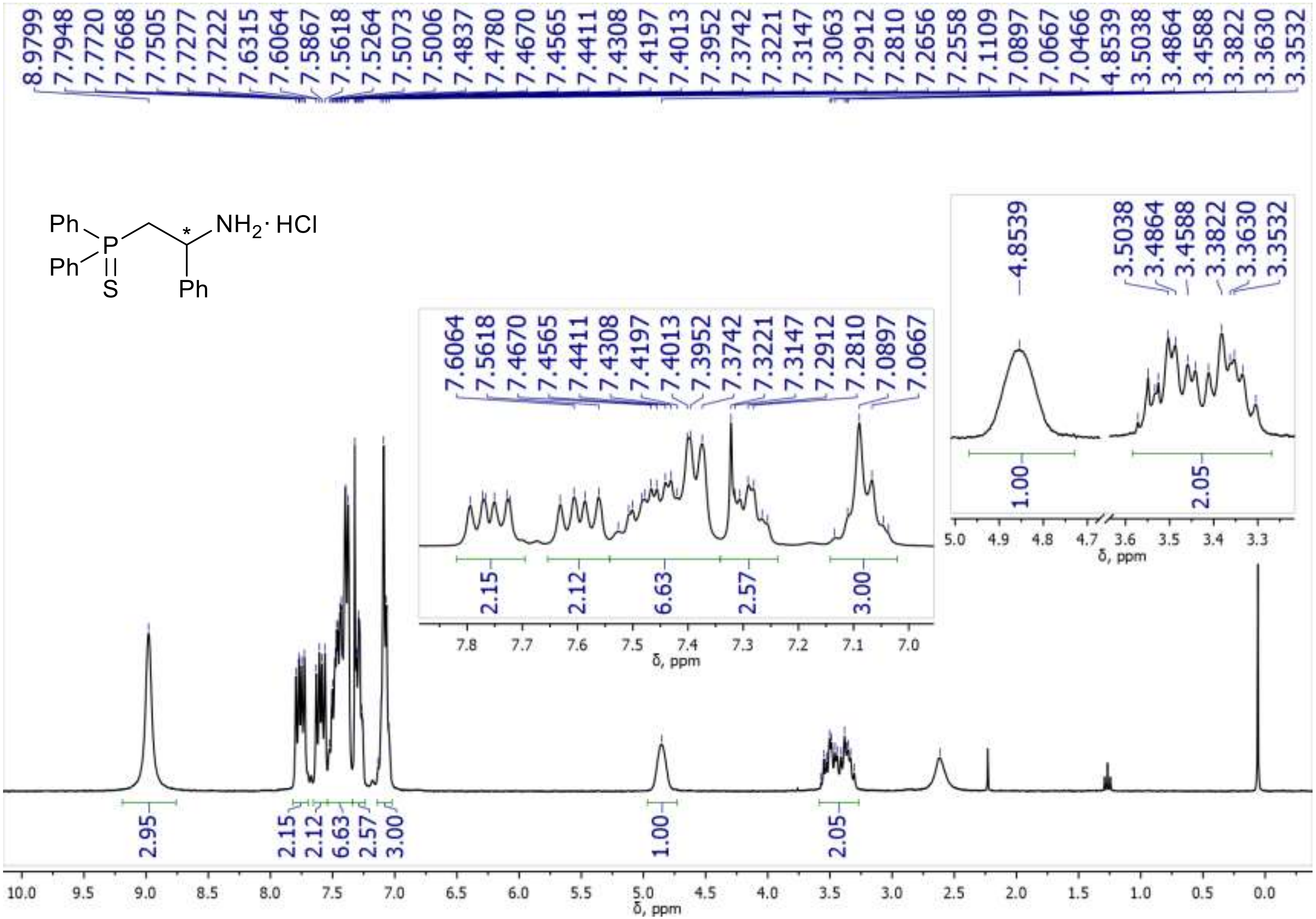

Figure S2. ^{1}H NMR spectrum of amine precursor **1a** (300.13 MHz, $CDCl_3$)

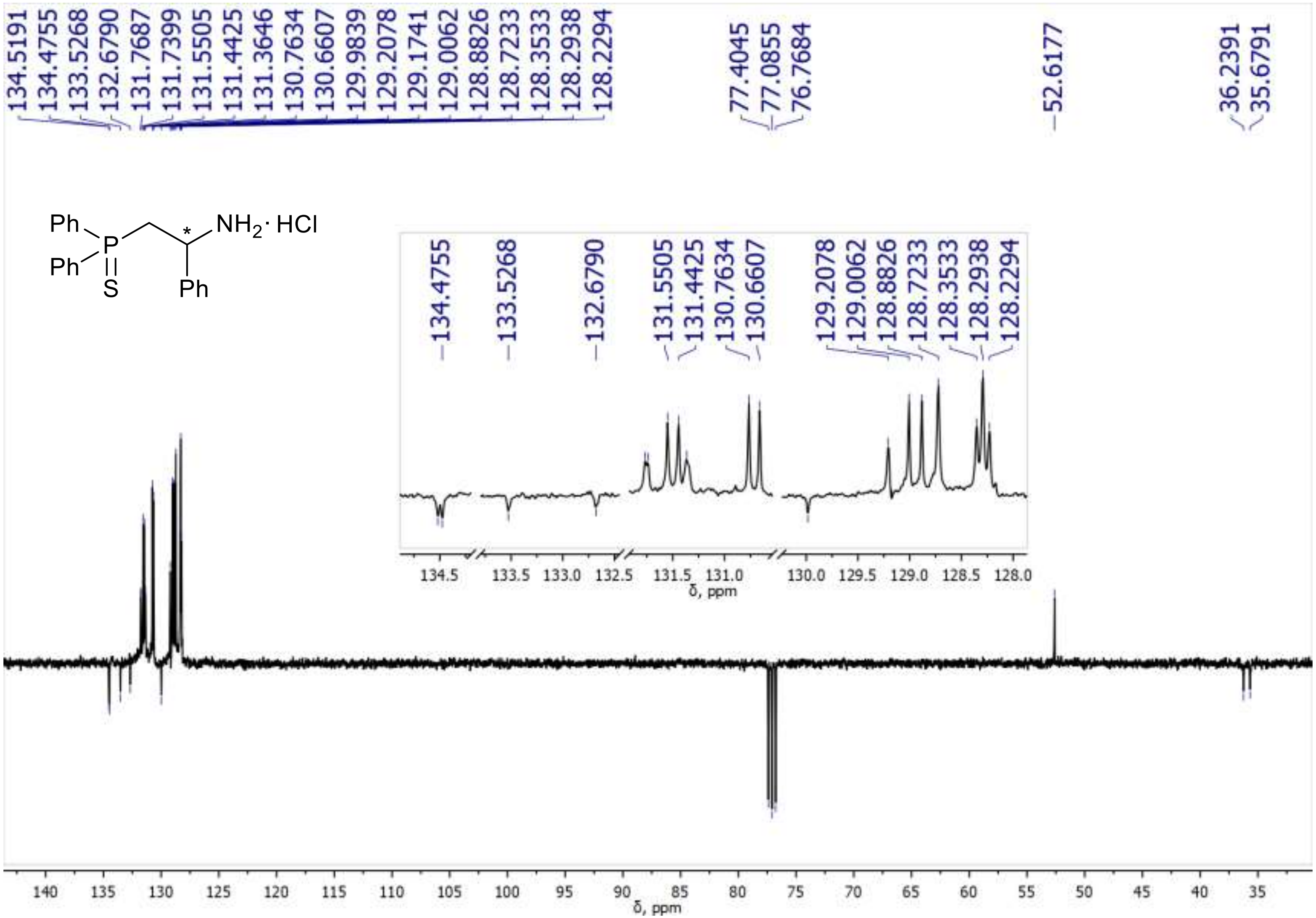

Figure S3. $^{13}C\{^{1}H\}$ NMR spectrum of amine precursor **1a** (100.61 MHz, $CDCl_3$)

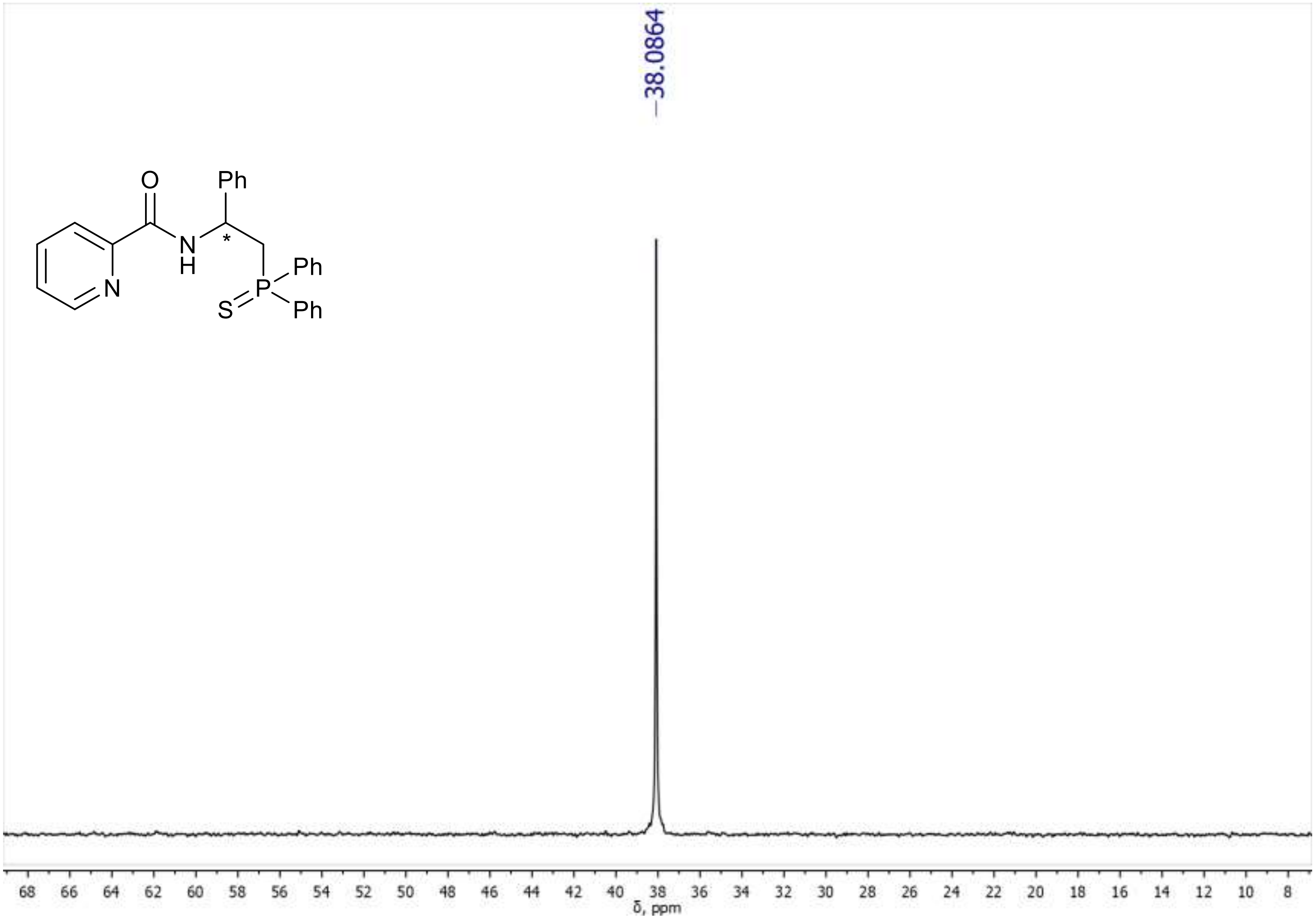

Figure S4. $^{31}P\{^{1}H\}$ NMR spectrum of ligand **2a** (121.49 MHz, $CDCl_3$)

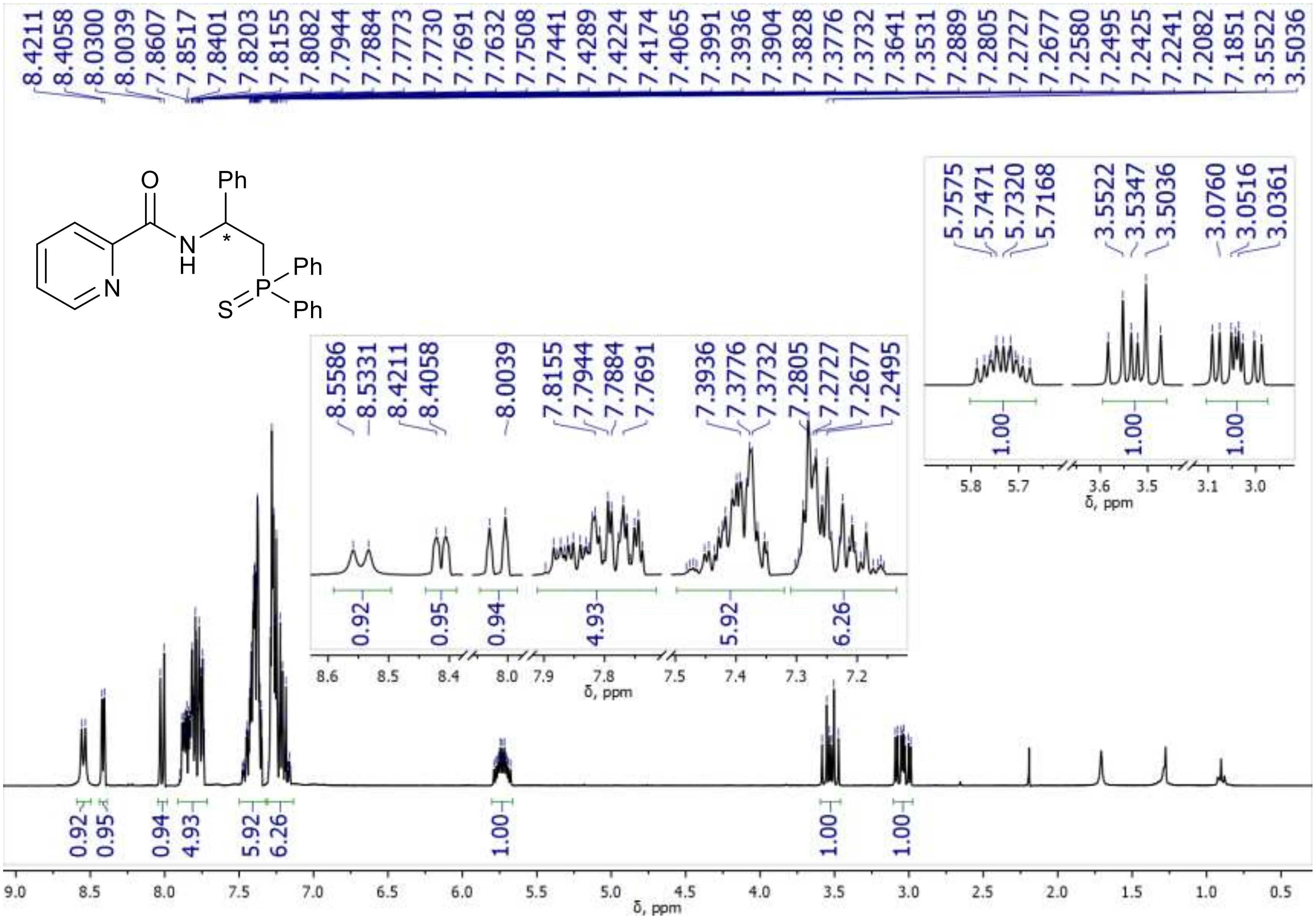

Figure S5. ^{1}H NMR spectrum of ligand **2a** (300.13 MHz, $CDCl_3$)

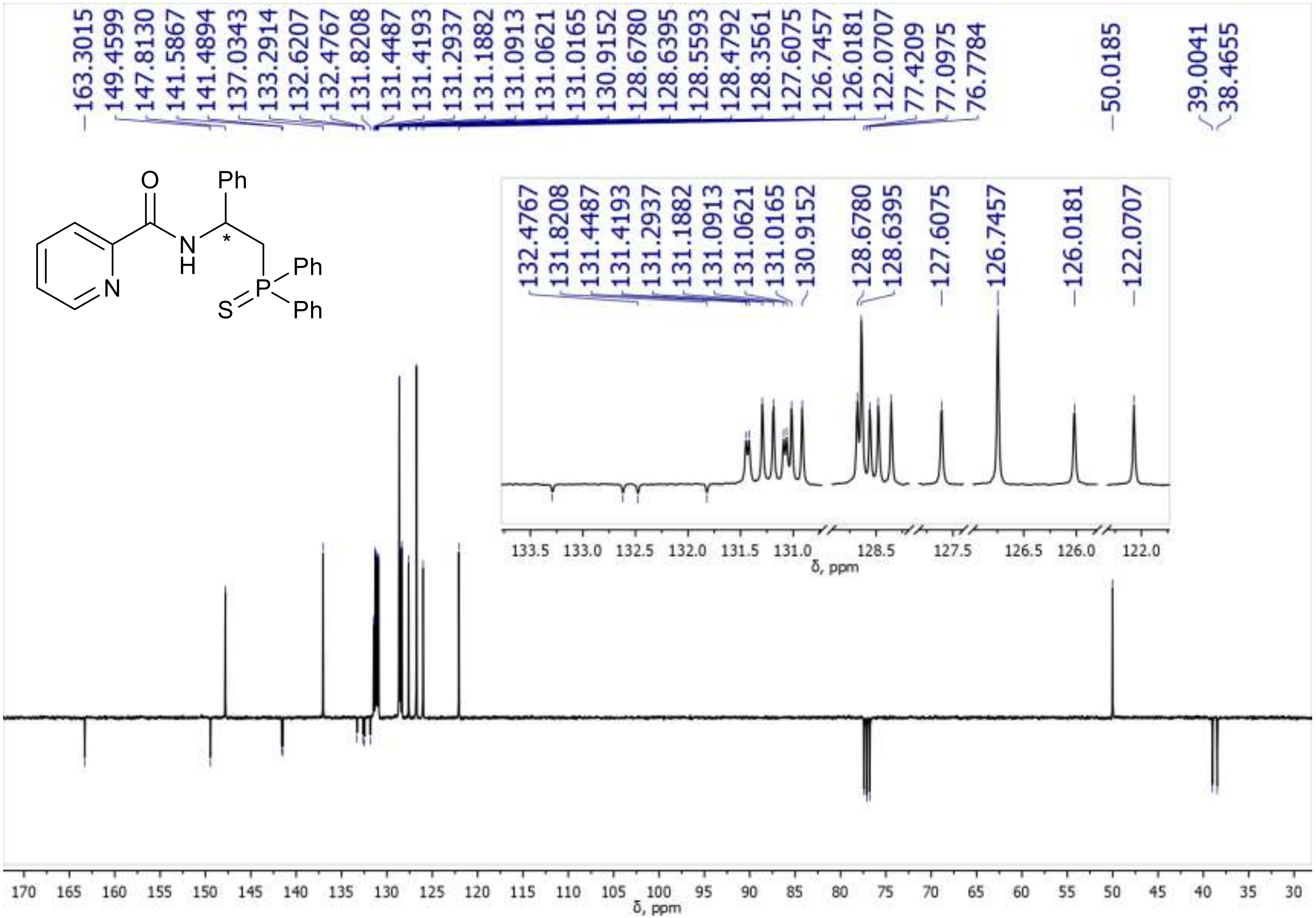

Figure S6. $^{13}C\{^1H\}$ NMR spectrum of ligand **2a** (100.61 MHz, $CDCl_3$)

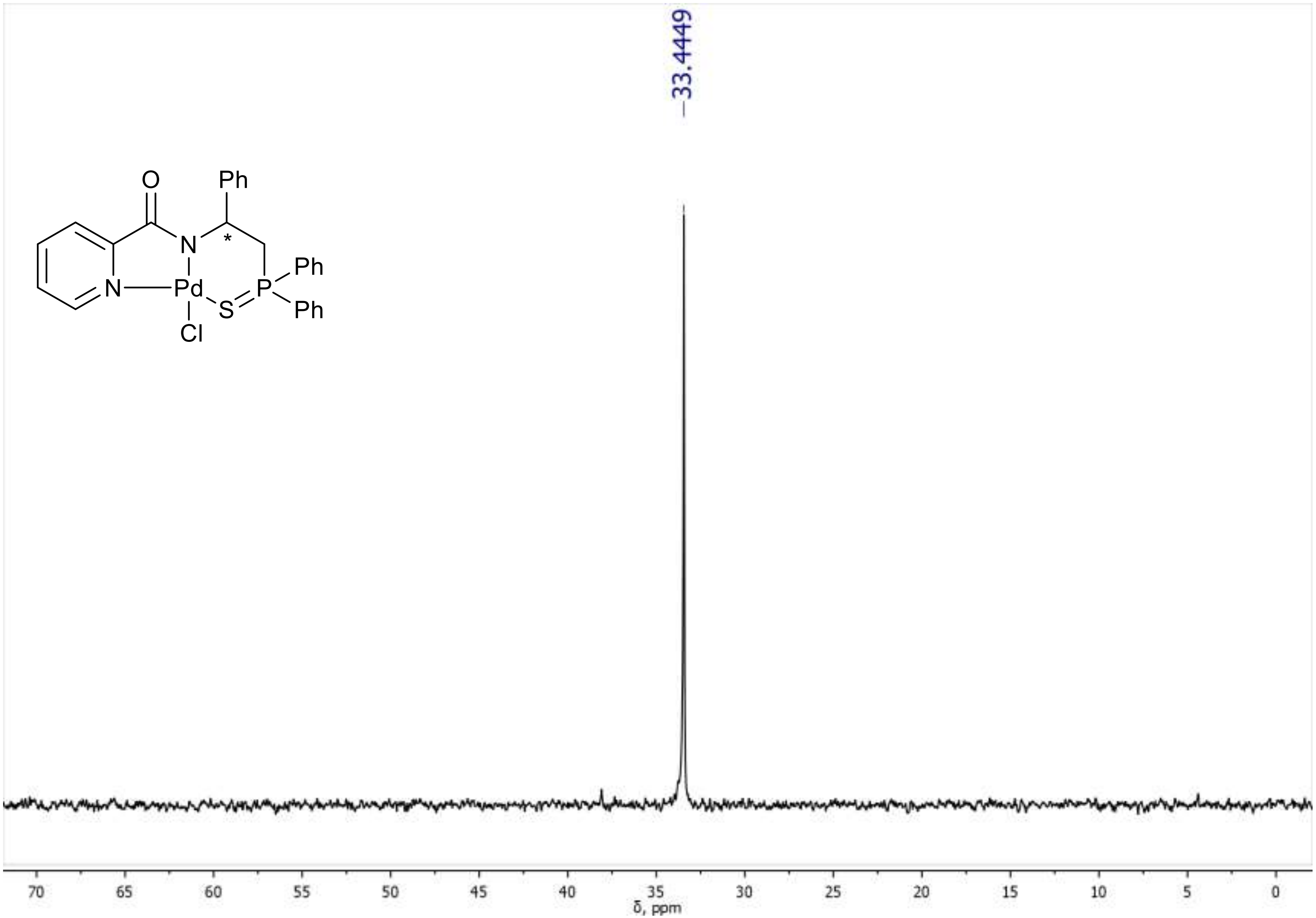

Figure S7. $^{31}P\{^{1}H\}$ NMR spectrum of complex **3a** (121.49 MHz, $CDCl_3$)

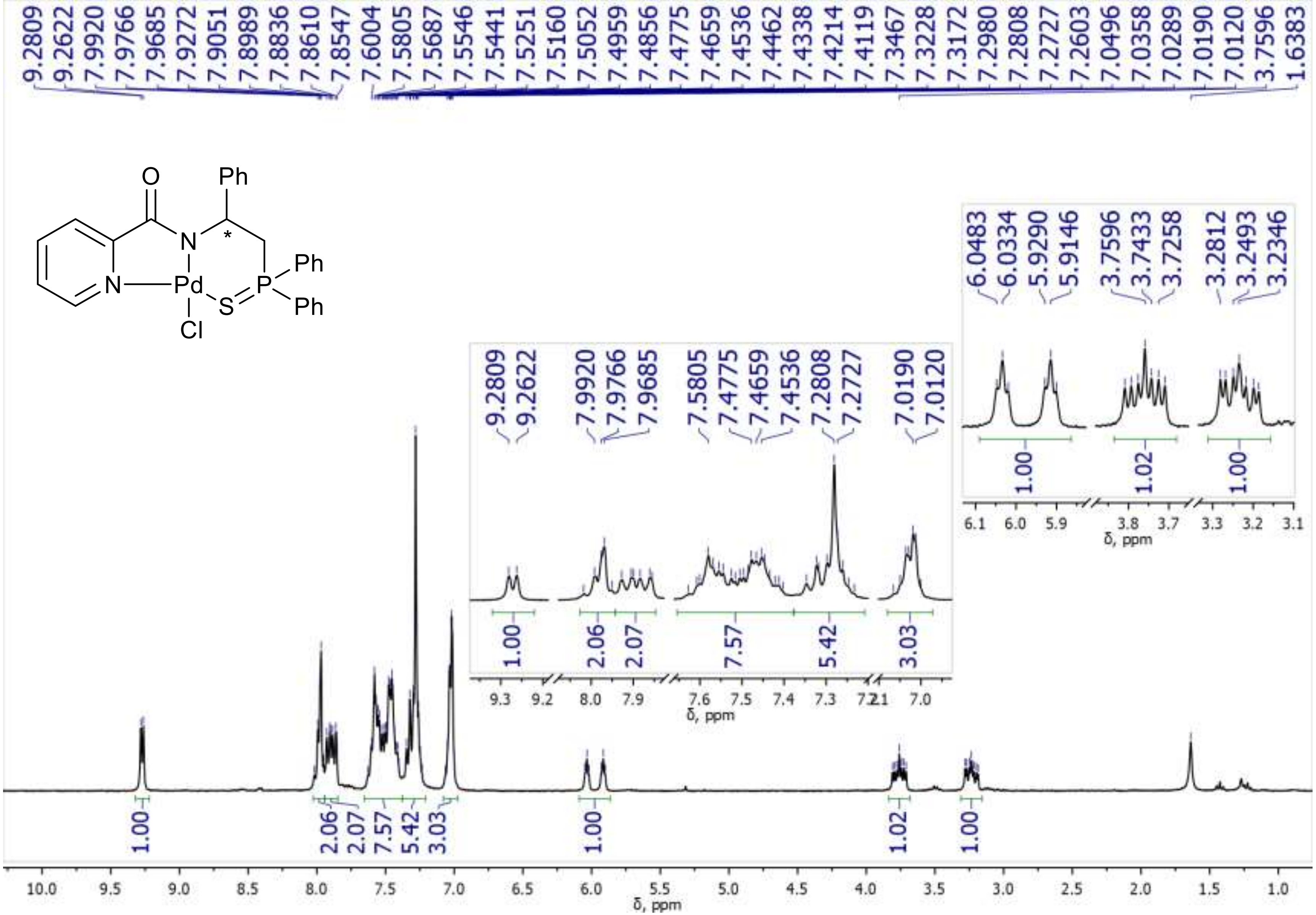

Figure S8. ^{1}H NMR spectrum of complex **3a** (300.13 MHz, $CDCl_3$)

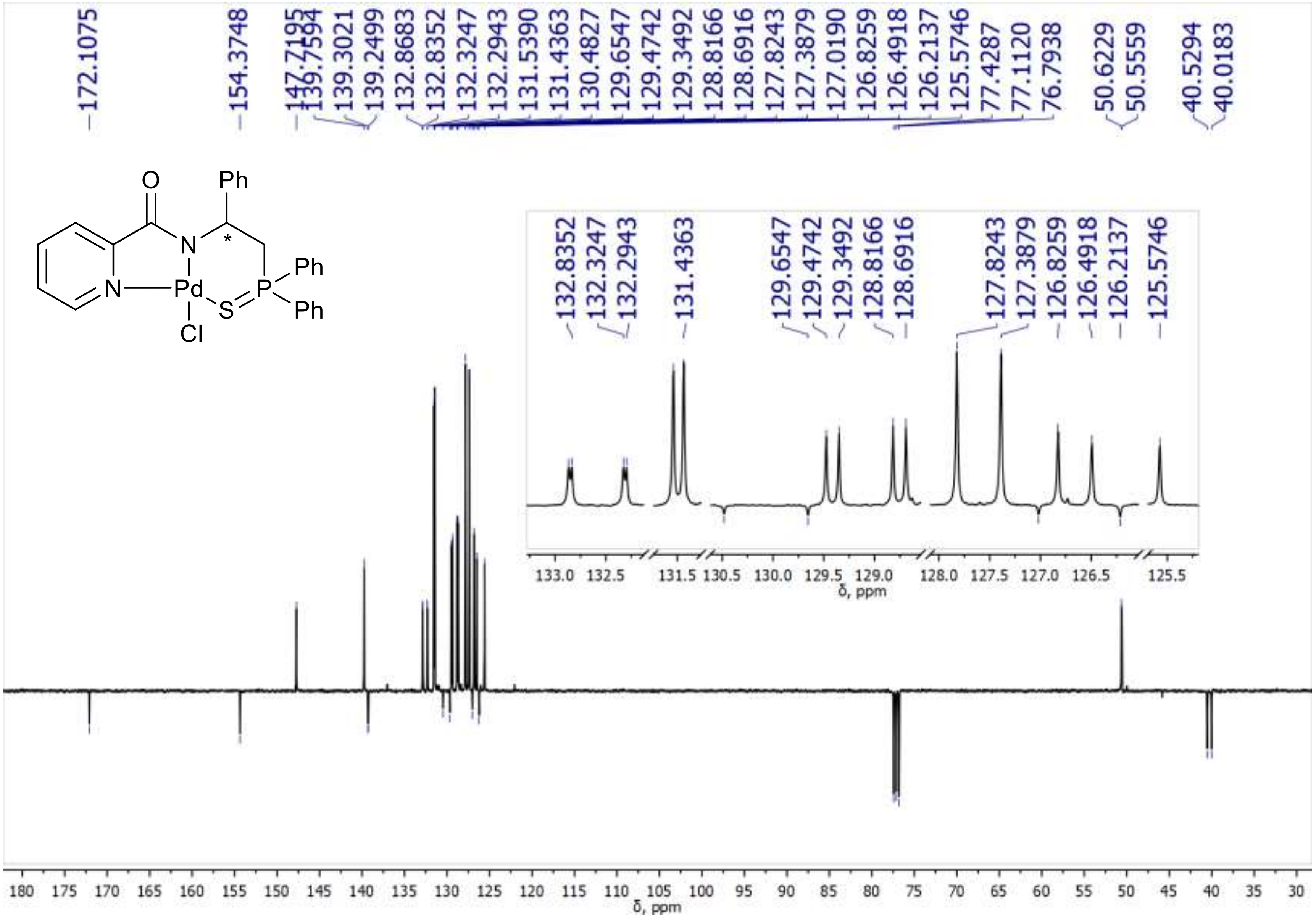

Figure S9. $^{13}C\{^{1}H\}$ NMR spectrum of complex **3a** (100.61 MHz, $CDCl_3$)

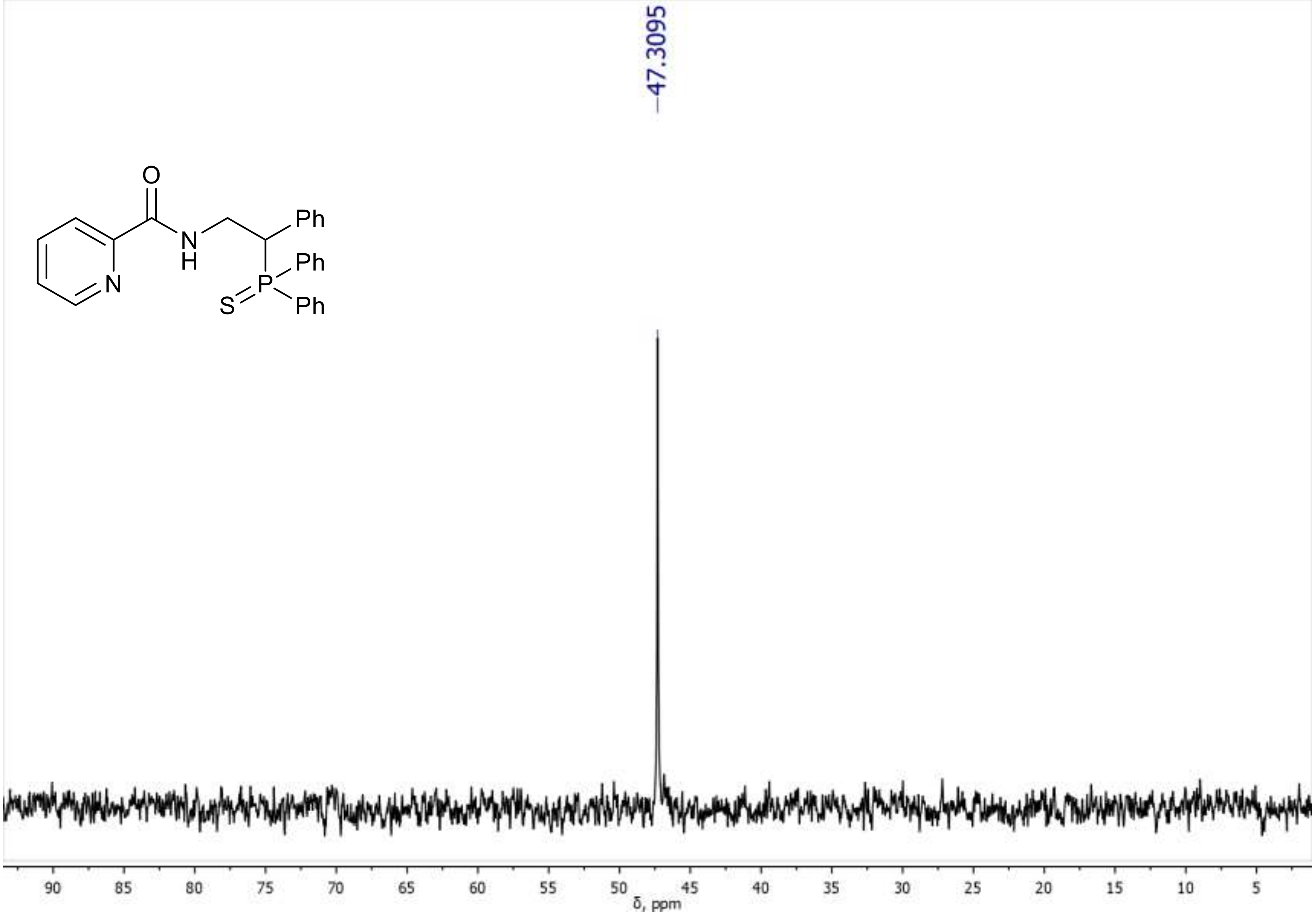

Figure S10. $^{31}P\{^{1}H\}$ NMR spectrum of ligand **6** (121.49 MHz, $CDCl_3$)

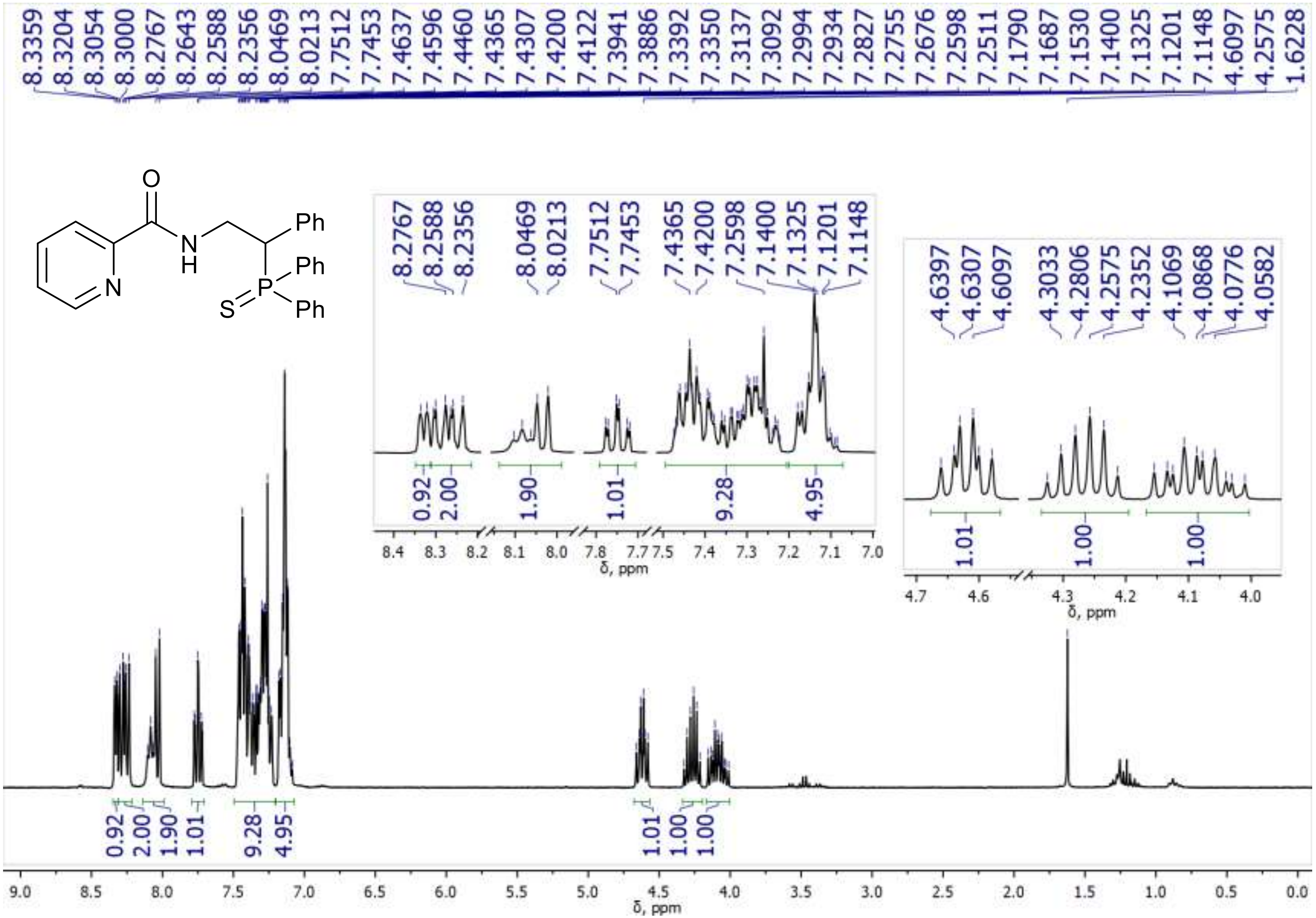

Figure S11. ^{1}H NMR spectrum of ligand **6** (300.13 MHz, $CDCl_3$)

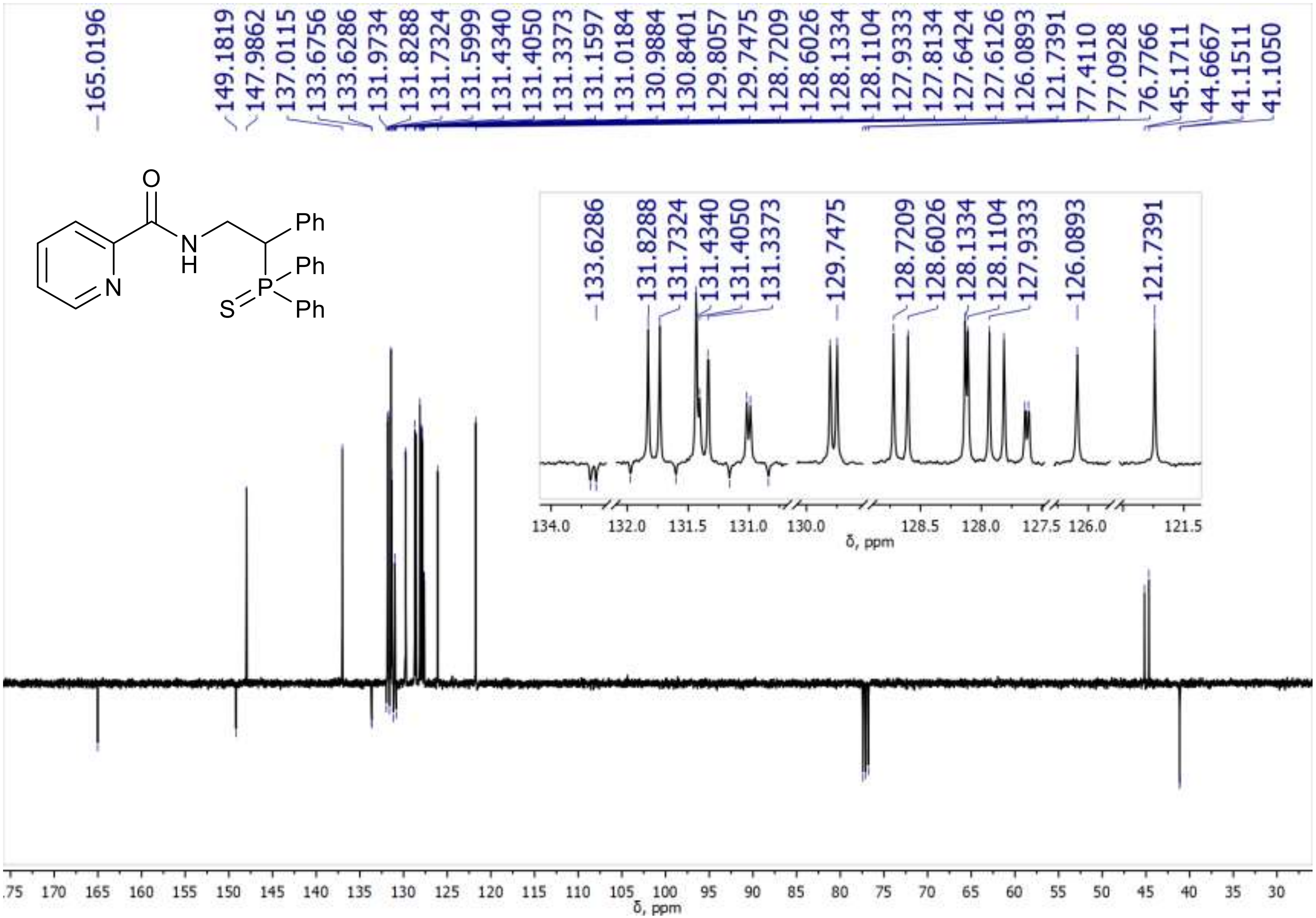

Figure S12. $^{13}C\{^{1}H\}$ NMR spectrum of ligand **6** (100.61 MHz, $CDCl_3$)

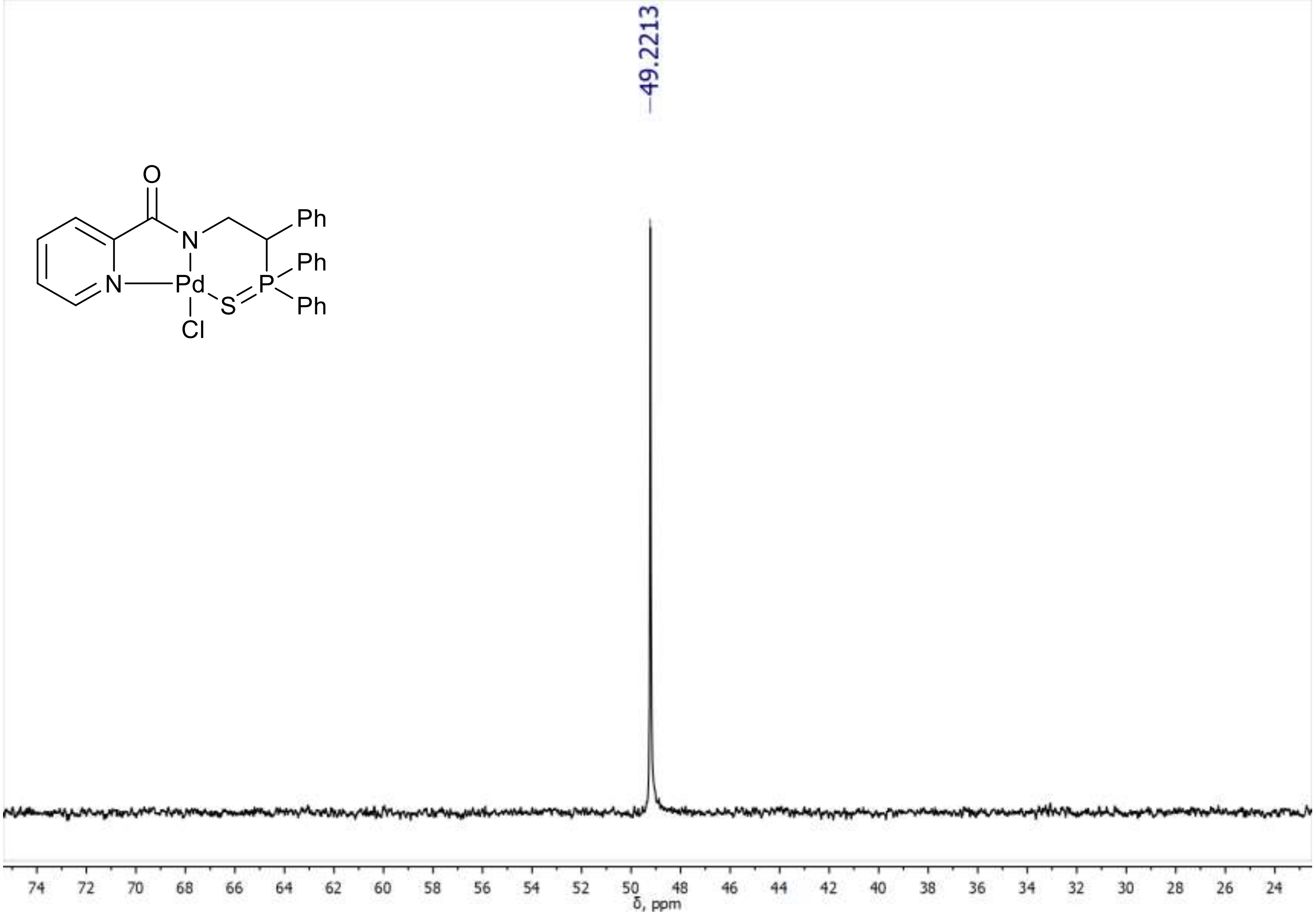

Figure S13. $^{31}P\{^{1}H\}$ NMR spectrum of complex **7** (121.49 MHz, $CDCl_3$)

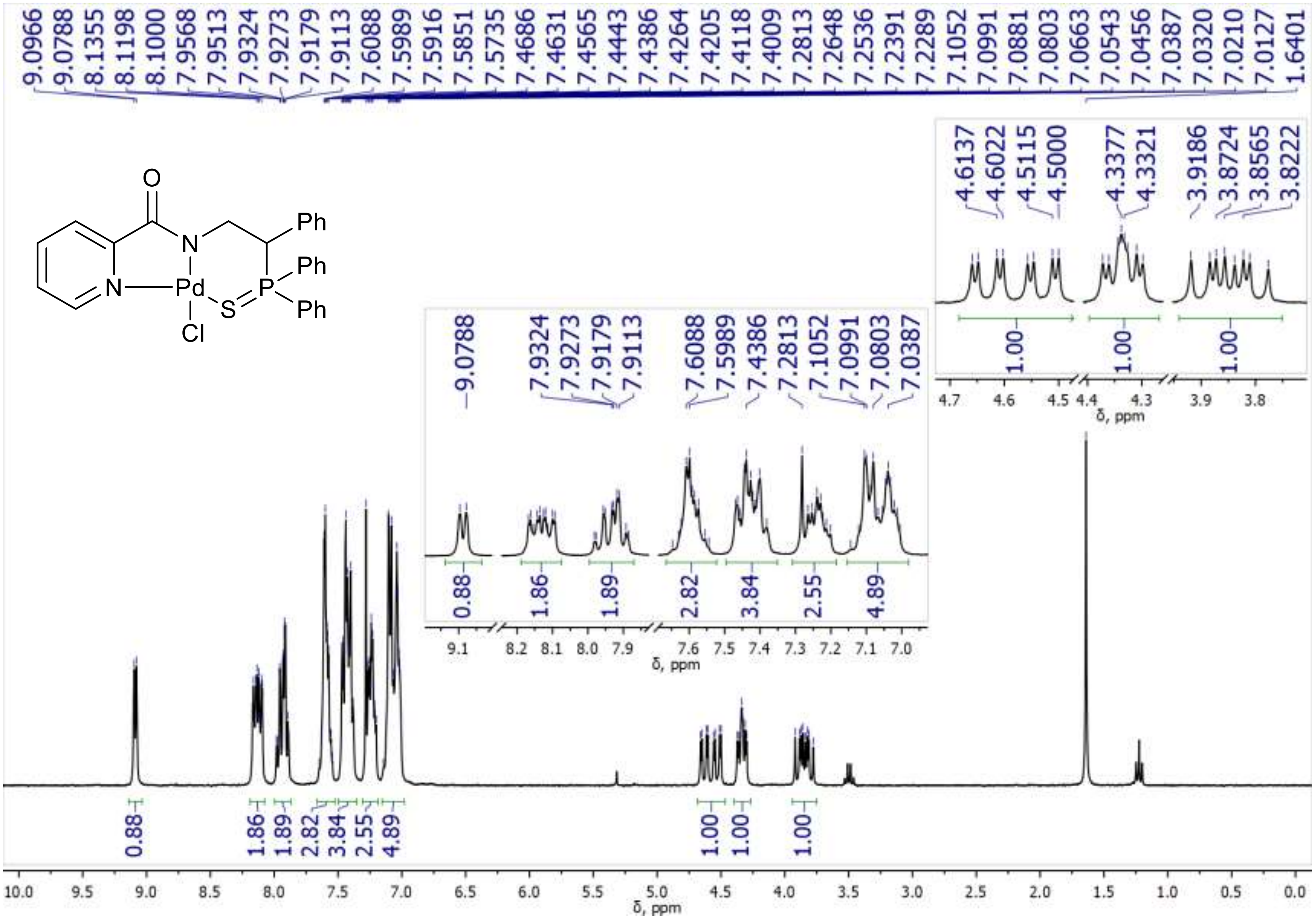

Figure S14. ^{1}H NMR spectrum of complex **7** (300.13 MHz, $CDCl_3$)

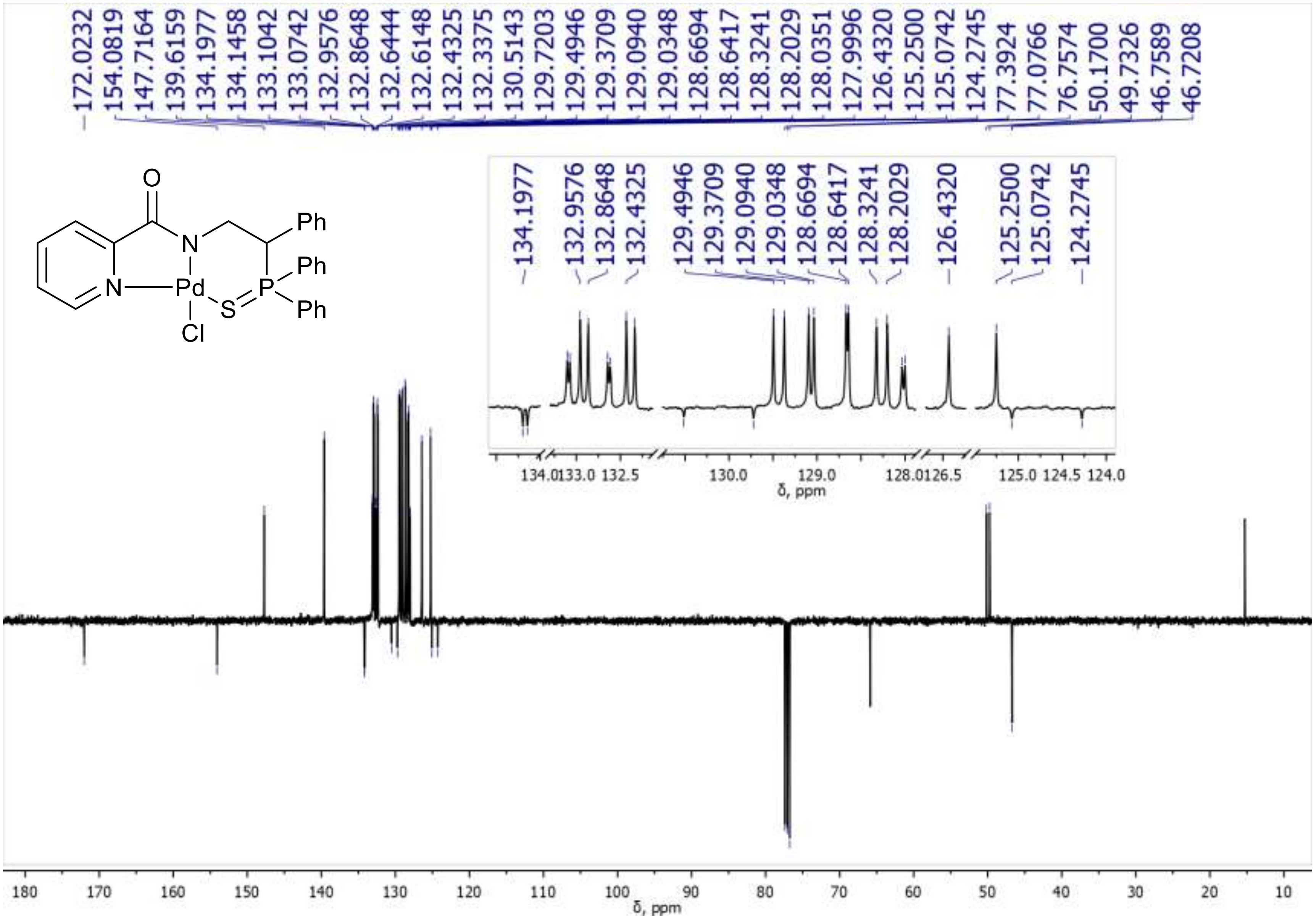

Figure S15. $^{13}C\{^{1}H\}$ NMR spectrum of complex **7** (100.61 MHz, $CDCl_3$)

Table S1. Primary antibodies used in the western blot analyses

Antibody name	Manufacturer	Catalog number	Dilution rate
Histone H2A.X (Ser139) (20E3) Rabbit mAb	Cell Signaling Technology	9718	1:1000
Rad51 (D4B10) Rabbit mAb	Cell Signaling Technology	8875	1:1000
Ku70 (D10A7) Rabbit mAb	Cell Signaling Technology	4588	1:1000
Ku80 Antibody	Cell Signaling Technology	2753	1:1000
beta-Actin (C4) Mouse mAb HRP	Santa Cruz Biotechnology	sc-47778 HRP	1:500
